# Sec61 complex/translocon: The role of an atypical ER Ca^2+^-leak channel in health and disease

**DOI:** 10.3389/fphys.2022.991149

**Published:** 2022-10-06

**Authors:** Jan B. Parys, Fabien Van Coppenolle

**Affiliations:** ^1^ Laboratory for Molecular and Cellular Signaling, Department of Cellular and Molecular Medicine & Leuven Kanker Instituut, KU Leuven, Leuven, Belgium; ^2^ CarMeN Laboratory, INSERM, INRA, INSA Lyon, Université Claude Bernard Lyon 1, Lyon, France; ^3^ Groupement Hospitalier EST, Department of Cardiology, Hospices Civils de Lyon, Lyon, France

**Keywords:** Sec61 complex/translocon, reticular Ca^2+^-leak channel, ER stress, cell survival, apoptosis, intracellular Ca^2+^ homeostasis

## Abstract

The heterotrimeric Sec61 protein complex forms the functional core of the so-called translocon that forms an aqueous channel in the endoplasmic reticulum (ER). The primary role of the Sec61 complex is to allow protein import in the ER during translation. Surprisingly, a completely different function in intracellular Ca^2+^ homeostasis has emerged for the Sec61 complex, and the latter is now accepted as one of the major Ca^2+^-leak pathways of the ER. In this review, we first discuss the structure of the Sec61 complex and focus on the pharmacology and regulation of the Sec61 complex as a Ca^2+^-leak channel. Subsequently, we will pay particular attention to pathologies that are linked to Sec61 mutations, such as plasma cell deficiency and congenital neutropenia. Finally, we will explore the relevance of the Sec61 complex as a Ca^2+^-leak channel in various pathophysiological (ER stress, apoptosis, ischemia-reperfusion) and pathological (type 2 diabetes, cancer) settings.

## 1 Introduction

Intracellular Ca^2+^ signaling forms the core of a ubiquitous signal transduction pathway controlling important cell biological processes, including fertilization, proliferation, development, learning and memory, muscle contraction, secretory behavior, metabolism, apoptosis, autophagy, etc. (Berridge et al., 2000; Bagur and Hajnóczky, 2017; Giorgi et al., 2018; Wacquier et al., 2019; Bootman and Bultynck, 2020). The activity of the various members of the so-called Ca^2+^-signaling toolkit and their mutual functional and/or structural interactions determine the spatio-temporal properties of the Ca^2+^ signals and thus their eventual physiological effect. This toolkit effectively consists of a plethora of Ca^2+^ pumps and exchangers, Ca^2+^ channels and Ca^2+^-binding proteins located in the cytosol, the plasma membrane or various organelles (Berridge et al., 2003).

The endoplasmic reticulum (ER) forms the largest intracellular Ca^2+^ store and the Ca^2+^ ions released from the ER play an important role in the occurrence of the above-mentioned complex spatio-temporal Ca^2+^ signals (Berridge, 2002; Lam and Galione, 2013). Members of two related families of Ca^2+^-release channels are expressed in the ER (and the SR, the sarcoplasmic reticulum), i.e. inositol 1,4,5-trisphosphate (IP_3_) receptors (IP_3_R) and ryanodine receptors (RyR). While the RyR is expressed in a predominant way in only a limited number of tissues, especially skeletal and cardiac muscle as well as the brain (Lanner, 2012), the IP_3_R is ubiquitously expressed (Foskett et al., 2007; Berridge, 2016; Prole and Taylor, 2019; Hamada and Mikoshiba, 2020). The latter are therefore considered the main ER Ca^2+^-release channels involved in intracellular Ca^2+^ signaling. To perform this function, they form large tetrameric structures (4 x ∼300 kDa) that are activated by IP_3_ and that additionally are exquisitely regulated by Ca^2+^ itself, by phosphorylation/dephosphorylation processes and by multiple protein-protein interactions (Foskett et al., 2007; Vanderheyden et al., 2009; Prole and Taylor, 2016; Prole and Taylor, 2019; Hamada and Mikoshiba, 2020).

Less well understood, though physiologically at least even important, are the so-called Ca^2+^-leak channels that, depending on the cell type and the intracellular milieu, modulate the basal permeability of the ER for Ca^2+^ (Camello et al., 2002; Sammels et al., 2010; Takeshima et al., 2015; Lemos et al., 2021). These Ca^2+^-leak channels can affect intracellular Ca^2+^ signaling in multiple ways (Lemos et al., 2021). First, even a very low level of leak activity will impact cell behavior in stress situations wherein the SERCA (sarco- and endoplasmic reticulum Ca^2+^ ATPase) pumps can no longer compensate for the Ca^2+^ leakage, thereby leading to decreased filling of the ER Ca^2+^ store. Second, if the latter occurs or if the endogenous leak activity is anyway exceeding the capacity of the SERCA pumps, the setpoint for the ER Ca^2+^ concentration ([Ca^2+^]_ER_) will lower and thus directly or indirectly lead to decreased IP_3_-dependent Ca^2+^ release. Finally, highly active Ca^2+^-leak channels may themselves produce small Ca^2+^ signals interfering in a positive or negative way with intracellular Ca^2+^ signaling.

Surprisingly, the Ca^2+^-leak channels form a large though very heterogenous group of proteins that share as their only common characteristic, their potency to increase the permeability of the ER membrane for Ca^2+^ (Lemos et al., 2021). Some of them are dysfunctional versions of proteins involved in physiological Ca^2+^ handling such as IP_3_R or RyR. Dysfunctions can originate from (excessive) post-translational modifications, protease-mediated cleavages or mutations. Ca^2+^ signaling events such as Ca^2+^ puffs and sparks can also be considered to result from Ca^2+^-leak activity, although the channels involved (IP_3_R and RyR, respectively) are not dysfunctional, and their activity strongly depend on the local environment, especially [Ca^2+^]_cyt_ and [Ca^2+^]_ER_ (Berridge, 2006; Cheng and Lederer, 2008; Konieczny et al., 2012). Even SERCA pumps can participate in Ca^2+^ leakage from the ER either by increased slippage of the pump (Inesi and de Meis, 1989) or when truncated (Chami et al., 2008). A second group of Ca^2+^-leak channels is formed by ion channels that are at least partially expressed in the ER, as some TRP, Orai and pannexin channels, members of the Bax-inhibitor 1 family or some less-studied proteins, such as mitsugumin 23. Finally, a third group consists of proteins that are well known for a physiological function that is absolutely unrelated to Ca^2+^ handling, but that have also been shown to function as Ca^2+^-leak channels.

The importance of some of the proteins of the latter group in Ca^2+^ handling has recently emerged (Lemos et al., 2021). Presenilins form a first example. Presenilins 1 and 2 are expressed in the ER and Golgi apparatus (Annaert et al., 1999). They represent the catalytic core of the γ-secretase complex, a protease involved in the cleavage of multiple proteins including the amyloid precursor protein (De Strooper et al., 1998). Presenilins have been shown to function as *bona fide* ER Ca^2+^-leak channels (Tu et al., 2006; Zatti et al., 2006; Bandara et al., 2013; Klec et al., 2019). However, other studies suggest that they regulate other Ca^2+^-handling proteins, e.g., IP_3_Rs (Shilling et al., 2012; Shilling et al., 2014). Of course, these two functions are not mutually exclusive. A second example, for which evidence of its role as a Ca^2+^-leak channel has accumulated over the years, is the Sec61 complex/translocon (hereafter called the ‘Sec61 complex’). Its primary function is of course related to protein import in the ER during translation (Lang et al., 2017; Gemmer and Förster, 2020), but its role in Ca^2+^ handling is increasingly evident and has recently also been linked to pathological situations. It will therefore be the subject of the present review.

## 2 Structure and role of the Sec61 complex in protein translation/translocation and in the ER-associated degradation process (ERAD)

### 2.1 Function of the Sec61 complex

In eukaryotic cells, some proteins must be translocated or inserted into the ER membrane. The precursors of these proteins are characterized by a signal peptide, a hydrophobic N-terminal sequence or a transmembrane helix of 20–30 amino acids (Simon and Blobel, 1991). The nascent polypeptide chain exits from the ribosome with its signal peptide. Its recognition by the signal recognition particle (SRP) causes elongation arrest (Grudnik et al., 2009). Subsequently, this ribosome-nascent chain complex is, in a GTP-dependent manner, targeted to the membrane via interactions between SRP and its receptor. After the ribosomes dock to the Sec61 complex, the signal peptide is released from the SRP, and the SRP dissociates from its receptor (for review, see Egea et al., 2005). Interestingly, protein translocation through the Sec61 complex is also intricately associated with the Sec62/Sec63 complex (Jung and Kim, 2021).

The transport of proteins in parallel with their translation, membrane insertion and processing via the Sec61 complex requires the coordinated action of different cofactors and enzymes. The functional core of the Sec61 complex in mammals is formed by three different subunits (α, β and γ), allowing the proteins to cross or insert into the ER membrane (for review, see Lang et al., 2017). This basic function is complemented by accessory components, which are physically associated with it (Grudnik et al., 2009; Dejgaard et al., 2010; Shen et al., 2012). These accessory components, including SPC (signal peptidase complex), OST (oligosaccharyltransferase), TRAM (translocating chain-associated membrane protein), Sec62/63 complex and TRAP (translocon-associated protein complex), assist the passage of proteins through the channel formed by the Sec61 complex, where they allow maturation of nascent chains by covalent modifications and their chaperone-like function (Haßdenteufel et al., 2018; Ichhaporia and Hendershot, 2021).

During translocation, nascent proteins are correctly folded by ER luminal chaperones. BiP (a Ca^2+^- and ATP-dependent HSP70 chaperone also named GRP78) is one of the major ER chaperones that assists nascent proteins in the folding process (Pobre et al., 2019). BiP is also involved in controlling Sec61 complex permeability during translocation (Lièvremont et al., 1997; Hamman et al., 1998; Schäuble et al., 2012; Hammadi et al., 2013). Other auxiliary proteins are also associated with the Sec61 complex, such as Sec63 (Müller et al., 2010; Lang et al., 2012; Conti et al., 2015) and ERj1 (Blau et al., 2005; Dudek et al., 2005). Calnexin, a lectin-type chaperone of the ER, allows the maturation and oligomerization of secretory glycoproteins, and may associate with the Sec61 complex core, at least transiently or in a substrate-specific manner (Chevet et al., 1999; Schnell and Hebert, 2003; Lakkaraju et al., 2012).

The tertiary structure of newly synthetized proteins is under the control of molecular chaperone proteins. Inappropriate folding and accumulation of misfolded proteins first trigger the unfolded protein response (UPR) leading to a decrease in protein synthesis and an increase in chaperone expression (Ma and Hendershot, 2001; Hwang and Qi, 2018; Zhang et al., 2019). In parallel, during ERAD, unfolded proteins are retrotranslocated from the ER to the cytoplasm, poly-ubiquitinated and degraded by the 26S proteasome (Needham et al., 2019). The main channel for retrograde transport of proteins during ERAD is formed by the multispanning ubiquitin ligase Hrd1 (Schoebel et al., 2017; Wu and Rapoport, 2018), although the Sec61 complex can also be involved (Römisch, 2017).

### 2.2 Architecture of the Sec61 complex

The transient nature of the many interactions at the Sec61 complex level made it difficult to determine its structure. Even in the case of the Sec61 complex core, the composition, architecture, and mechanism of operation remain difficult to determine. However, technical advances, particularly in the field of cryo-electron microscopy, have made it recently possible to increase the understanding of the heterotrimeric Sec61 complex.

Studies on human cells have shown that the main components of the Sec61 complex are Sec61 (α, β and γ), TRAP and OST (Pfeffer et al., 2014). A secondary structure can be defined at the subnanometer scale, including the protein-conducting channel (Pfeffer et al., 2015). It has not yet been clearly defined whether OST transiently associates with the Sec61 complex for a specific phase of translocation or whether different types of Sec61 complexes coexist in the cell. It has, however, been shown that initiation of translation strengthens the association between OST and Sec61 (Conti et al., 2015; Bai et al., 2018), which tends towards the first hypothesis. However, no such conclusive pattern could be revealed *in vivo* (Mahamid et al., 2016).

#### 2.2.1 The pore

The main feature of the ER Sec61 complex is the existence of an aqueous pore across the membrane. Thanks to experiments using puromycin treatment, the Sec61 complex has been seen as a channel gated by the ribosome. Indeed, these ER transmembrane pores were detected by conductivity measurements, and the dependence of these channels on puromycin suggested that the nascent polypeptide chain must be released from the ribosome to allow the transmembrane passage of ions (Simon and Blobel, 1991). Since the channel is closed when the ribosomes are detached from the membrane, the passage of ions through the channel is dependent on both the nascent chain and the ribosome (Simon and Blobel, 1991; Van Coppenolle et al., 2004).

Since the lumen of the ER, and not the cytoplasm, forms a continuum with the Sec61 complex pore upon protein translocation, the ribosome itself must form the permeability barrier by strongly binding the cytoplasmic surface of the Sec61 complex and sealing the pore from there (Crowley et al., 1994).

The experiments conducted by Hamman *et al.* (Hamman et al., 1998) indicate that the ribosome remains in place on the Sec61 complex until the translation is complete. At this stage, the pore of the Sec61 complex linked to the ribosome had a diameter of 40–60 Å, potentially allowing Ca^2+^ leakage. In contrast, the pore of the Sec61 complex has an internal diameter of 9–15 Å when not bound to the ribosome (Hamman et al., 1997).

#### 2.2.2 The components of the Sec61 complex

##### 2.2.2.1 Sec61

**FIGURE 1:**
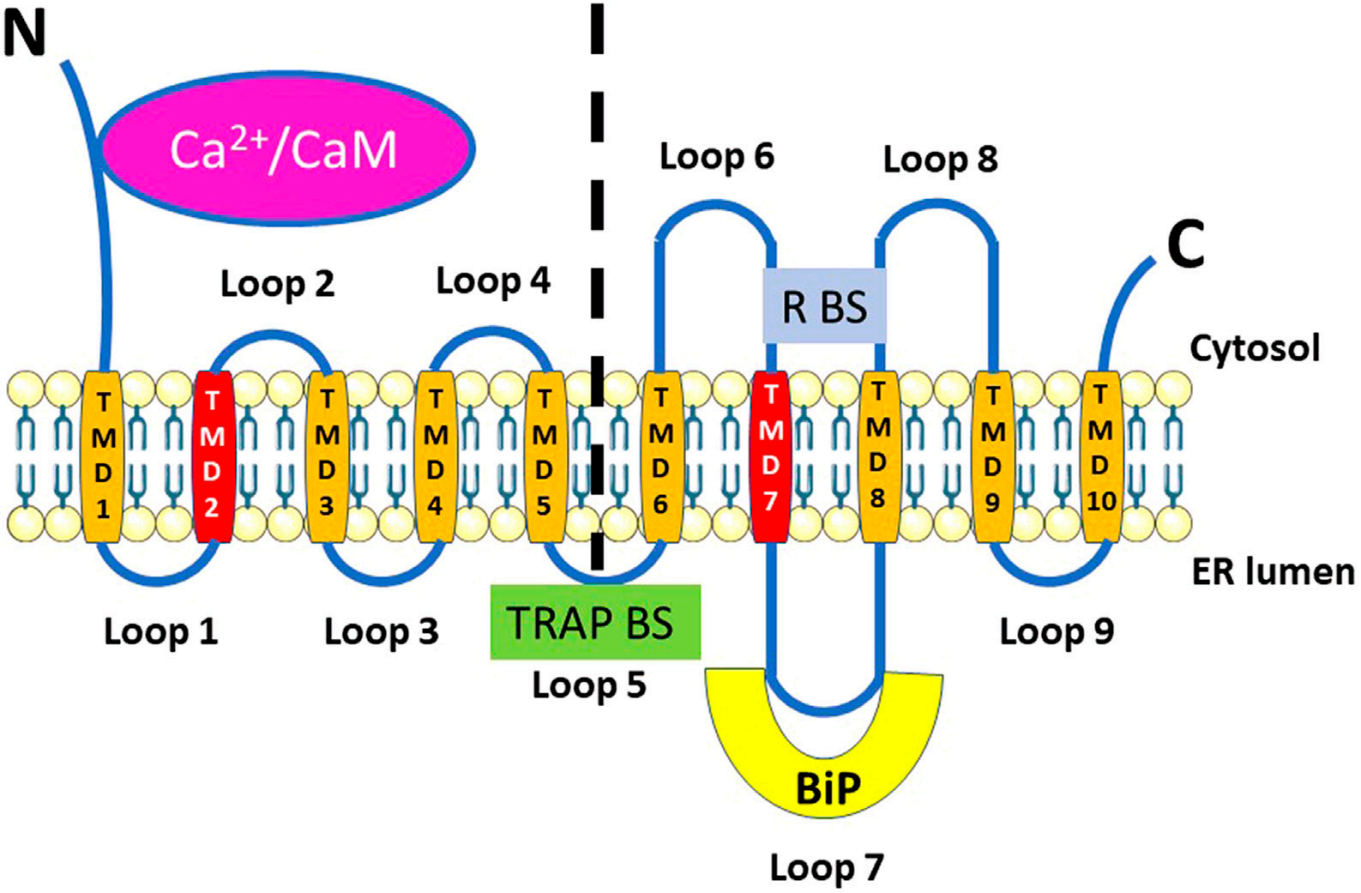
Structure of Sec61α, highlighting elements relevant for its function as ER Ca^2+^-leak channel. Sec61α is characterized by having 10 transmembrane domains (TMD1-10, in orange) arranged in two pseudo-symmetrical N- and C-terminal halves (delineated by the vertical dashed line) with both the N and C termini located in the cytosol. The two sterically adjacent TMD2 and TMD7 (in dark orange) form the lateral gate. The interaction of Ca^2+^/calmodulin (Ca^2+^/CaM) at the N-terminus and of BiP at loop 7 is shown. In addition, the luminal binding site for the translocon-associated protein complex (TRAP BS) on loop 5, at the interphase between the N- and C-terminal halves, and of the ribosome (R BS) on loops 6 and 8 are indicated.

Sec61 has a heterotrimeric structure forming a channel allowing the passage of proteins (Figure 1). The Sec61α subunit consists of 10 transmembrane helices, arranged in two pseudosymmetrical N- and C-terminal halves around a central pore, impermeable to passive ion flow. The Sec61β and Sec61γ subunits are located at the periphery of the Sec61 complex, and each has a transmembrane helix (Berg et al., 2004; Egea and Stroud, 2010; Li et al., 2016).

During protein transport and translation, the ribosome binds to cytosolic loops 6 and 8 of Sec61α (Figure 1). A recent mechanistic model shows that Sec61 transiently opens to allow insertion of the transmembrane helix or nascent protein signal sequence into the ER membrane while remaining impermeable to low-molecular-weight proteins (Becker et al., 2009; Gogala et al., 2014; Park et al., 2014; Voorhees et al., 2014; Voorhees and Hegde, 2016).

The pore of the Sec61 complex formed by Sec61 completely encompasses the ER lipid bilayer through which the newly synthesized secretory proteins are translocated (Swanton and Bulleid, 2003). The heteromeric Sec61 complex forms the nucleus of this pore, which can be blocked by BiP on the luminal side. The ribosome binds to the cytosolic side, and the Sec61 complex can be considered an SRP-ribosome gated channel.

##### 2.2.2.2 TRAP

TRAP is a heterotetrameric membrane protein complex, the role of which is to assist Sec61 with protein insertion and more specifically in the topogenesis of polytopic membrane proteins (Sommer et al., 2013). It is made up of three subunits (α, β, δ) forming a transmembrane helix and a luminal domain, while TRAPγ forms a set of four transmembrane helices with a small cytosolic domain (von Heijne and Gavel, 1988). The luminal part of TRAP binds Sec61α at the level of the two N- and C-terminal halves (Figure 1) and could thus influence the conformation of Sec61α and interact with the newly synthesized proteins that are translocated. Interestingly, in TRAPβ-depleted HeLa cells, thapsigargin induced via the Sec61 complex a smaller increase in [Ca^2+^]_cyt_ than in control cells (Nguyen et al., 2018). It would therefore be interesting to compare in further experiments simultaneously [Ca^2+^]_cyt_ and [Ca^2+^]_ER_ to decipher whether the absence of TRAPβ decreases the Ca^2+^ leak and/or partly depletes the Ca^2+^ stores.

##### 2.2.2.3 OST

The OST complex is made up of at least seven proteins. OST catalyzes the glycosylation of newly synthesized proteins (Kelleher and Gilmore, 2006). One of the subunits of OST is STT3. It occurs in two paralogous forms STT3A and STT3B, which are involved in glycosylation during and after translation respectively (Ruiz-Canada et al., 2009). They are associated with at least six accessory subunits of OST, the function or structure of which are not yet completely understood: ribophorin I, ribophorin II, OST48, DAD1, N33 (or IAP) and OST4. The N33 subunit of OST has oxidoreductase activity and is believed to increase glycosylation efficiency by slowing the conformational changes of glycoproteins (Mohorko et al., 2014). Recent findings using high-resolution cryoelectron microscopy revealed that OST binds to Sec61α and partially penetrates the lumen of the ER (Braunger et al., 2018; Ramírez et al., 2019). However, its interaction with the Sec61 complex has yet to be further determined.

##### 2.2.2.4 Other important ER proteins

Calnexin, an intraluminal ER membrane protein acting as a chaperone during nascent protein conformation (Chen et al., 1995), may be linked to nascent polypeptide chains (Chen et al., 1995; Oliver et al., 1996; Tatu and Helenius, 1997). Calnexin therefore appears to be close to the Sec61 complex, but there is as yet no evidence of its participation in the complex.

Further ER proteins such as calreticulin, protein disulfide isomerase, BiP and ERp57 also transiently interact with the nascent protein chain (Nicchitta and Zheng, 1997; Oliver et al., 1997; Tatu and Helenius, 1997), but it was arbitrarily decided to consider only membrane proteins associated with the Sec61 complex.

#### 2.2.3 The ribosome

Sec61α is located in the center of the Sec61 complex and binds the ribosome via its loops 6 and 8, close to the exit peptide of the ribosome (Raden et al., 2000; Song et al., 2000). While TRAP specifically binds a region of Sec61α, OST interacts with Sec61 via a larger interface in the ER membrane. On the cytosolic face of the Sec61 complex, the small cytosolic domains of TRAP and OST interact with the large ribosomal subunit via specific cytosolic contact sites (Silberstein and Gilmore, 1996; Wang and Dobberstein, 1999; Kriegler et al., 2020; Mohanty et al., 2020).

## 3 Sec61 complex, Ca^2+^ leakage and pharmacology

As some antibiotics inhibit translation by acting on the Sec61 complex, these compounds turned out to be very useful to modulate Sec61 complex opening, either inducing or inhibiting Ca^2+^ leakage. Nevertheless, it is important to note that acute pharmacological modulation of Sec61 complex opening with those antibiotics during short-term Ca^2+^-imaging experiments is too brief to significantly affect protein translation/translocation (Van Coppenolle et al., 2004). Indeed, these molecules act on the Ca^2+^ permeability of the Sec61 complex in the order of minutes, while they will take a longer time to act on protein translation in a detectable way. Similarly, during long-term treatment of cells with these compounds it is necessary to pay attention to use a concentration that is able to modify Sec61 complex permeability to Ca^2+^ without significantly affecting protein translation.

### 3.1 Three states for one complex

There are three known conformations of Sec61α: idle, intermediate and open. In its non-translating state, Sec61α binds to the ribosome with a plugged gate corresponding to the idle conformation (Voorhees et al., 2014). Ca^2+^ leakage via the Sec61 complex probably does not occur in this state (Wirth et al., 2003). After binding to the ribosome and just before signal peptide engagement, Sec61α moves toward the intermediate state (Bhadra et al., 2021). Here, the Sec61α complex “breathe” along the opened lateral gate (Li et al., 2016), which may trigger Ca^2+^ permeability. Once nascent protein translocation occurs through the channel, Ca^2+^ cannot cross the pore. After translocation, when the peptidic chain has been released, the Sec61 complex remains transiently in an open post-translocation state that allows Ca^2+^ leakage via the aqueous pore. Indeed, the time between the ribosome detaches from the Sec61 complex, and the reformation of a luminal plug corresponds to the time period during which Ca^2+^ leakage can occur through the wide-open pore with a 50 Å diameter. Subsequently, the Sec61 complex returns again to the idle state when the ribosomes come off (Bhadra et al., 2021).

### 3.2 Pharmacological aspects

Puromycin is a structural analog of phenylalanyl-tRNA, causing the formation of an abnormal peptidyl-puromycin. The newly synthetized polypeptide chains are therefore incomplete, which causes their detachment from the Sec61 complex and the inhibition of protein synthesis (Traut and Monro, 1964). At this level, the ribosome is still linked to the Sec61 complex (Pestova and Hellen, 2001; Van Coppenolle et al., 2004), and the pore is open, allowing Ca^2+^ leakage (Van Coppenolle et al., 2004; Wonderlin, 2009).

Concerning pactamycin, its mode of action is still debated. During translation, all tRNA substrates attach to three ribosome binding sites named A (aminoacyl), P (peptidyl) and E (exit). These three sites are located at the junction between the ribosomal subunit and the tRNA (Yusupov et al., 2001). Indeed, the first studies showed that pactamycin associates with the ribosome, thereby preventing tRNA from entering the P site. This would be due to a change in the conformation of the ribosome or by direct competition, thus inhibiting protein translation (Eida and Mahmud, 2019). Regardless, the peptidic chain is released in the presence of pactamycin during translation, allowing Ca^2+^ leakage (Al-Mawla et al., 2020).

Anisomycin is a translation inhibitor that acts by binding to a part of the 60S subunit (Hummel et al., 1987; Rodriguez-Fonseca et al., 1995) of the ribosome that has been suggested to be the peptidyl transferase center (Rodriguez-Fonseca et al., 1995; Iordanov et al., 1997). Anisomycin has been shown to inhibit puromycin reaction in an *in vitro* system (Ioannou et al., 1998). The inhibition of the peptidyl transferase activity by anisomycin prevents elongation of the peptide chain and any Ca^2+^ leakage through the Sec61 complex (Van Coppenolle et al., 2004; Hammadi et al., 2013).

Emetine interacts with the 40S ribosome subunit at the E site, inhibiting the progression of the ribosome on mRNA. This molecule is therefore an irreversible inhibitor of translation elongation, which stabilizes the ribosome/nascent chain complex, causing the pore of the Sec61 complex to close (Al-Mawla et al., 2020). It should be noted that emetine also seems to mobilize Ca^2+^ from the Golgi apparatus, although the Ca^2+^ channel involved was not identified (Gallegos-Gómez et al., 2018). The latter point should be taken into consideration when using the compound.

Cycloheximide is commonly used for control purposes. This antibiotic acts as an inhibitor of elongation, binding the E site of the ribosome without any modulation of Sec61 complex permeability to Ca^2+^ (Van Coppenolle et al., 2004).

It is now also possible to modulate the permeability of the Sec61 complex with molecules other than antibiotics. Indeed, recently, the *Mycobacterium ulcerans* exotoxin mycolactone has been shown to enhance ER Ca^2+^ leakage via the Sec61 complex (Bhadra et al., 2021). The mechanisms of action of mycolactone on the Sec61 complex are detailed in Section 3.5.

Another molecule of interest is eeyarestatin, a chemical inhibitor of protein degradation via the ERAD system (Wang et al., 2008; Wang et al., 2009) and of translocation of nascent polypeptides into the ER via the Sec61 complex (Cross et al., 2009). Recently, eeyarestatin analogs have also been found to mediate ER Ca^2+^ leakage via the Sec61 complex (Gamayun et al., 2019). In this interesting study, eeyarestatin was proposed to bind Sec61α in the open state, where it would prevent the closure of the lateral gate, keeping Sec61α in a Ca^2+^-permeable state.

The list of drugs acting on the Ca^2+^ permeability of the Sec61 complex is gradually increasing, thereby widening the scope of studies in this field.

### 3.3 The Sec61 complex: A new functional, ER Ca^2+^-leak channel

As mentioned above (see Section 2.2.1), the pore of the Sec61 complex is the largest in the ER, with a diameter of 40–60 Å in the ribosome-bound state and a smaller diameter of 9–15 Å in the ribosome-free state (Hamman et al., 1997; Hamman et al., 1998). Subsequently, the role of the Sec61 complex as an ER Ca^2+^-leak channel emerged thanks to the work of Wonderlin’s group showing that small polarized molecules could cross the ER membrane through the Sec61 complex (Heritage and Wonderlin, 2001; Roy and Wonderlin, 2003). We therefore hypothesized that Ca^2+^ ions would also move across the ER membrane via the Sec61 complex. To verify this hypothesis, the main experimental problem was how to best open the Sec61 complex pore to directly measure ER Ca^2+^ leakage. Similar to Wonderlin and collaborators, we started by using puromycin to, for the first time, directly detect Ca^2+^ leakage through the Sec61 complex in mouse pancreatic acinar cells (Lomax et al., 2002).

In subsequent experiments, we continued the investigation of the cell physiological role of the Sec61 complex as an ER Ca^2+^ leak channel. Using human cancerous prostate cells (the LNCaP cell line), we determined that puromycin induced Ca^2+^ leakage through the Sec61 complex independently of IP_3_Rs or RyRs (Van Coppenolle et al., 2004). Moreover, for this study, we systematically used anisomycin to counteract puromycin’s action to control the specificity of action of these compounds on the Sec61 complex. In a similar way, other studies used pactamycin instead of puromycin and emetine instead of anisomycin (Ong et al., 2007; Al-Mawla et al., 2020). The fundamental cell physiological role of the Sec61 complex was elucidated in a second study using LNCaP cells (Flourakis et al., 2006). The [Ca^2+^]_ER_ at rest is the result of the equilibrium between Ca^2+^ uptake mediated by the SERCA pumps and passive Ca^2+^ leakage through Ca^2+^-leak channels. Inhibition of SERCA pumps by the specific inhibitor thapsigargin therefore fully reveals the passive Ca^2+^ release through ER Ca^2+^-leak channels, as it is not compensated by reuptake. The measured Ca^2+^ leak is, in those conditions, the result of all the ER Ca^2+^-leak channels open at this stage. This leak appeared to be mostly mediated by the Sec61 complex. Moreover, similar results were obtained using EGTA-AM (a cytosolic Ca^2+^ chelator) to enhance Ca^2+^ release. Altogether, these data highlight the role of the Sec61 complex as a major Ca^2+^-leak channel in the LNCaP cell model. In addition, it is well known that ER Ca^2+^ depletion can activate SOCs (store-operated channels). Interestingly, we have also demonstrated that Ca^2+^ release that occurs via the Sec61 complex activates SOCs. This was the first characterization of store-operated Ca^2+^ entry triggered by passive Ca^2+^-leak channels (Flourakis et al., 2006).

Giunti *et al.* elegantly studied the efflux of Ca^2+^ from ER-derived rat liver microsomal vesicles (Giunti et al., 2007). They detected two basal passive pathways for Ca^2+^ efflux. One of them is carried by the Sec61 complex since the leakage was stimulated by puromycin perfusion. The second efflux pathway is more mysterious and requires counterion influx. It does not involve inactive SERCA pumps, Bcl-2 proteins, or known Ca^2+^ channels such as IP_3_Rs or RyRs. Interestingly, the authors also noticed that the efflux was largest in the rough microsomal subfractions, which are enriched in the Sec61 complex. Thanks to this approach, this work provides strong evidence for the role of the Sec61 complex as a functional ER Ca^2+^-leak channel at the organellar level.

In addition to these studies, we should mention the interesting work of Layhadi *et al.* (Layhadi and Fountain, 2017). They highlighted in THP-1 macrophages the role of the Sec61 complex in the control of the [Ca^2+^]_cyt_ at rest. In the absence of extracellular Ca^2+^, inhibition of the Sec61 complex with anisomycin reduced the resting [Ca^2+^]_cyt_. In addition, the authors demonstrated that inhibition of the Sec61 complex by anisomycin enhances the response to ADP (via P2Y receptors) in human primary macrophages. Altogether, this work clearly indicates that the Sec61 complex is not only an ER Ca^2+^-leak channel but also acts, at least in macrophages, as a regulator of ER Ca^2+^ content and of the [Ca^2+^]_cyt_ at resting state.

Taken together, various studies have demonstrated that the Sec61 complex is a functional ER Ca^2+^-leak channel in several cell types. Nevertheless, the Ca^2+^-leakage rate depends on the dynamics of plug binding/dissociating the pore. An important question, therefore, is which factors will control Ca^2+^ leakage?

### 3.4 Regulation of Sec61 complex Ca^2+^ leakage from the ER by Ca^2+^-binding proteins

A permanently open ER Ca^2+^-leak channel would in the long run form a hazard for the cell as both ER Ca^2+^ levels and cytosolic Ca^2+^ levels control many aspects of cell function, including the occurrence of ER stress and/or the induction of cell death (Orrenius et al., 2003; Pinton et al., 2008; Krebs et al., 2015; La Rovere et al., 2016; Danese et al., 2021). It can therefore be expected that intra-ER and/or cytosolic proteins sensing the local [Ca^2+^] control the Ca^2+^ flux through the Sec61 complex.

#### 3.4.1 The regulating role of BiP

As stated above (see Section 2.1), BiP is an important ER intraluminal heat shock protein. It has a chaperone function towards newly synthesized proteins, controls the protein flux through the Sec61 complex, and activates the UPR in ER stress conditions (Pobre et al., 2019). Moreover, it participates in the regulation of intracellular Ca^2+^ homeostasis by acting as an intraluminal Ca^2+^-binding protein (Lièvremont et al., 1997), by stabilizing the type 1 IP_3_R (Higo et al., 2010) and by controlling ER Ca^2+^ leakage through the Sec61 complex (Schäuble et al., 2012; Hammadi et al., 2013). During ER stress, BiP preferentially targets accumulating unfolded or misfolded proteins, thereby releasing the three canonical ER-stress sensors, inositol-requiring enzyme 1 (IRE1), protein kinase RNA-like ER kinase (PERK), and activating transcription factor 6 (ATF6), which consequently become free to activate the UPR (Ron and Walter, 2007). In addition, during ER stress, BiP will also dissociate from both the IP_3_R and the Sec61 complex, leading to complex changes in ER Ca^2+^ handling. This includes first a decreased Ca^2+^ release from the ER and subsequently an increased Ca^2+^ release that can lead to cell apoptosis (Kiviluoto et al., 2013).

In relation to the Sec61 complex, BiP is an allosteric effector of the Sec61 channel, supporting the translocation of proteins through it (Lang et al., 2017). A BiP-binding site on a minihelix located in loop 7 of Sec61α (Figure 1) appears to be involved in the gating of the Sec61 complex and in the regulation of ER Ca^2+^ leakage through the Sec61 complex (Schäuble et al., 2012; Schorr et al., 2015). The expression in human HeLa cells of the Sec61α-Y344H mutant deficient in BiP binding (see Section 4.1) increased ER Ca^2+^ leakage (Schäuble et al., 2012). In the same study, an increased Ca^2+^ leak was also observed in conditions in which BiP was made unavailable, either via siRNA-mediated gene silencing or via induction of protein misfolding by short-term treatment with dithiothreitol or tunicamycin. This effect could not be mimicked by other chaperones, such as protein disulfide isomerase, calreticulin or GRP94, indicating the specificity of BiP compared to other chaperones. However, in this role, BiP is assisted by two of its intraluminal co-chaperones of the Hsp40 family, ERj3 and ERj6. siRNA-induced depletion of HeLa cells of either one of these two proteins led to increased Sec61-mediated Ca^2+^ leakage out of the ER (Schorr et al., 2015). This regulation was specific for ERj3 and ERj6 as depleting the Hsp40 proteins ERj1, Sec63 (also known as ERj2), ERj5 and ERj7 had no such effect.

**FIGURE 2:**
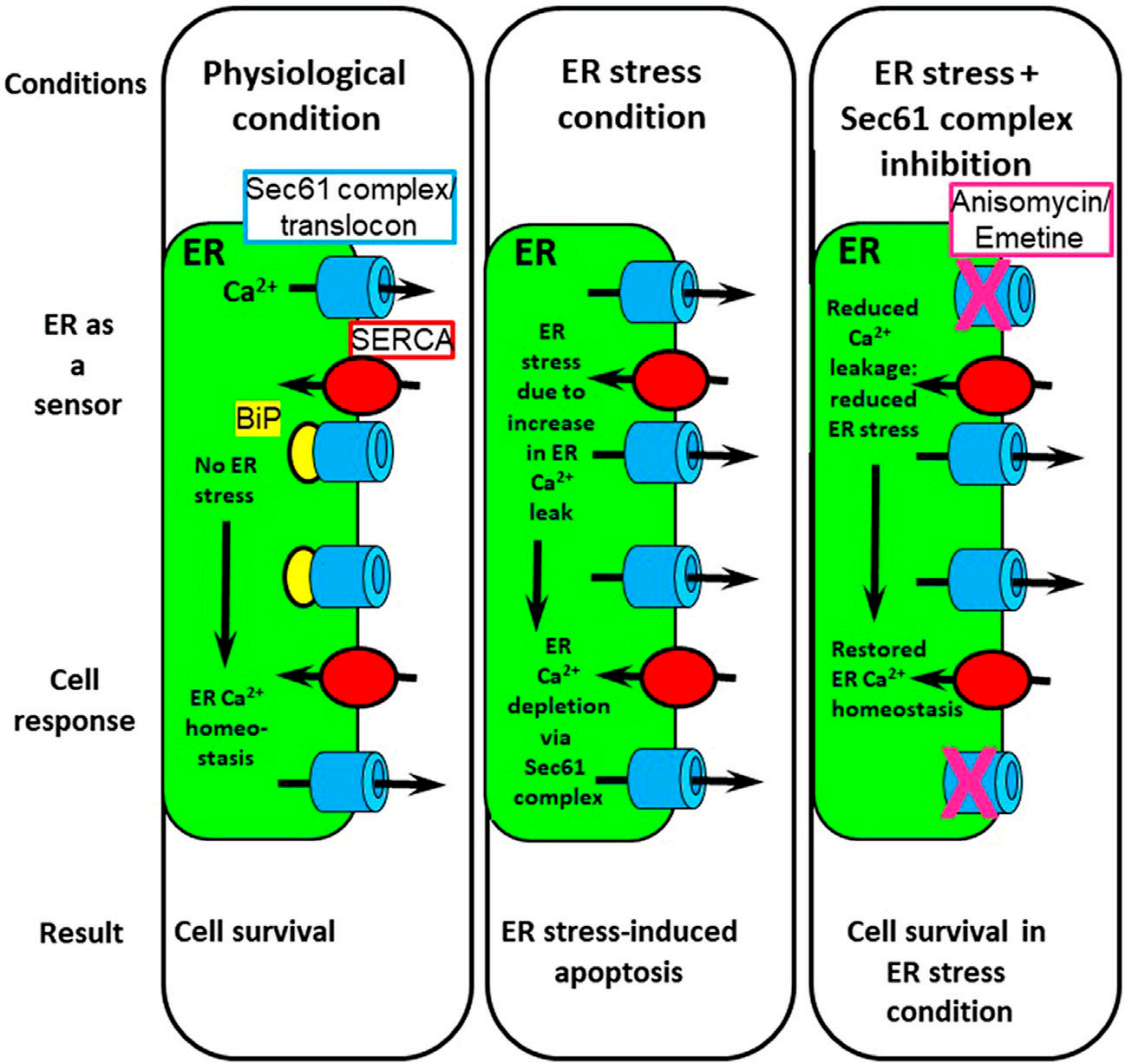
Involvement of the Sec61 complex/translocon in cell survival. In physiological condition, ER Ca^2+^ homeostasis is due to a balance between Ca^2+^ leak and Ca^2+^ re-uptake by SERCA pumps. ER stress triggers Ca^2+^ depletion of the ER Ca^2+^ stores via an increase in Ca^2+^ leakage through the Sec61 complex. Pharmacological inhibition of the Sec61 complex with anisomycin or emetine during ER stress restores ER Ca^2+^ homeostasis and protects cells from apoptosis. Adapted from Cassel et al., Plos One, 2016 (Cassel et al., 2016) licensed under a Creative Commons Attribution 4.0 International License.

The control of ER Ca^2+^ leakage by BiP has interesting functional consequences for the cell. First, as the presence of unfolded or misfolded proteins results in the release of BiP from the Sec61 complex, cells with a particularly high protein synthesis rate (e.g., β cells of the pancreas or hepatocytes) are particularly prone to experience increased Ca^2+^ leakage out of the ER, potentially leading to apoptosis (Hammadi et al., 2013; Kiviluoto et al., 2013; Schorr et al., 2015; Lang et al., 2017; Lemos et al., 2021) (Figure 2). In addition, since BiP is dependent on ATP for its function, it was proposed (Lang et al., 2019) that low [ATP]_ER_ will lead to decreased BiP activity and thus increased Ca^2+^ leakage out of the ER. The subsequent decrease in [Ca^2+^]_ER_ and increase in [Ca^2+^]_cyt_ will then activate the ADP/ATP exchanger of the ER (Klein et al., 2018) to replenish ER ATP content.

In conclusion, BiP, the luminal plug of the Sec61 complex, might attach and detach from the pore with particular dynamics determining the Ca^2+^-leakage rate depending on physiological cell conditions (such as ER stress, BiP expression or rate of translation).

#### 3.4.2 The regulatory role of calmodulin (CaM)

CaM is a ubiquitously expressed cytosolic Ca^2+^-binding protein. It contains four EF-hand motifs with an affinity for Ca^2+^ in the physiological range (Kd’s between 0.5 and 5 µM), making CaM ideally suited as an intracellular Ca^2+^ sensor (Chin and Means, 2000). CaM therefore modulates a plethora of proteins, including various Ca^2+^ pumps and channels.

It is thus particularly interesting that Ca^2+^ leakage through the Sec61 complex is also modulated by CaM (Erdmann et al., 2011). Sec61α contains in its N-terminal domain a high-affinity binding site (a.a. 19–32) that specifically interacts with Ca^2+^/CaM but does not interact with apoCaM (Figure 1). Electrophysiological experiments determined that CaM induced Ca^2+^-dependent closure of the Sec61 channel. The reverse also holds, as treatment with the CaM antagonists ophiobolin A or trifluoperazine enhanced Ca^2+^ leakage. Moreover, molecular modeling suggested that Ca^2+^/CaM binds in the gap between the ribosome and the Sec61 complex.

### 3.5 Modulation of Sec61-mediated Ca^2+^ leakage by bacterial toxins

Mycolactone is an exotoxin produced by *Mycobacterium ulcerans* that causes Buruli ulcer, a chronic skin necrosis (Demangel et al., 2009; Sarfo et al., 2016; Demangel and High, 2018). Pre-ulcerative and ulcerative lesions are painless, often delaying curative actions (En et al., 2008). *Mycobacterium ulcerans* infections lead to the suppression of the immune system allowing the multiplication of bacteria in the skin (Yotsu et al., 2018).

Many molecular targets of mycolactone have been identified, including the Sec61 complex (Baron et al., 2016; Morel et al., 2018; Gérard et al., 2020; Demangel, 2021; O’Keefe et al., 2022). Mycolactone inhibits Sec61-dependent protein translation/translocation into the ER (Hall et al., 2014). This has been linked to the mechanism of immunosuppression induced by mycolactone via inhibition of T cell activation and antigen presentation (Baron et al., 2016; Grotzke et al., 2017). Since the Sec61 complex is a target of mycolactone, the question arose as to the potential effects of this toxin on ER Ca^2+^ permeability. This aspect has been studied by Bhadra *et al.* (Bhadra et al., 2021). Using the HEK293 and HCT116 cell lines, they demonstrated that mycolactone enhances ER Ca^2+^ leakage via the Sec61 complex. The absence of Ca^2+^ depletion in cells expressing Sec61α mutants, resistant to mycolactone binding, corroborates the direct action of the toxin on the complex. Interestingly, they postulate that among the three known conformations of Sec61α, idle, intermediate and open (see Section 3.1), mycolactone stabilizes the intermediate conformation, locking the Sec61 complex in a Ca^2+^-permeable conformation.

### 3.6 The regulatory role of Sec62

Evidence exists that for post-translational protein translocation, the Sec61 complex associates with the Sec62/Sec63 complex. Sec62 is a 399 a. a. protein characterized by having both its N- and C-terminal regions in the cytosol, flanking the two transmembrane domains (TMD) connected by a short intraluminal loop (Daimon et al., 1997; Tyedmers et al., 2000).

Analysis of various prostate cancer cell lines demonstrated that reduced Sec62 protein levels lead to larger Ca^2+^ leakage out of the ER and reduced cell viability while increased Sec62 expression correlated with protection against thapsigargin-induced apoptosis (Greiner et al., 2011). These results strongly suggest that Sec62 is an inhibitor of ER Ca^2+^ leakage, most likely via a direct or indirect action on the Sec61 complex. Subsequent work indicated that Ca^2+^ affected the binding of the C terminus of Sec62 to the N terminus of Sec61α (Linxweiler et al., 2013). Moreover, mutation of a putative Ca^2+^-binding motif (a.a. 308–319) in the C terminus of Sec62, increased thapsigargin-induced Ca^2+^ leakage out of the ER. Taking into account that Sec62 silencing and treatment with CaM antagonists lead to the same phenotype, the authors propose a model in which Sec62 binding to Sec61 is relieved by Ca^2+^, allowing Ca^2+^/CaM to occupy its binding site and thus to inhibit ER Ca^2+^ leakage through the Sec61 complex. Obviously, another possible mechanism would involve the recruitment of BiP at the luminal side, subsequent to Sec62/Sec63 binding.

Taken together, the regulation of Sec61 complex-mediated Ca^2+^ leakage by multiple Ca^2+^-dependent proteins may indicate the importance of keeping Ca^2+^ leakage under tight control, or may indicate that depending on the situation, e.g., the presence or absence of ER stress, the cell relies on a different mechanism for controlling Ca^2+^ leakage out of the ER.

## 4 Involvement of Sec61 complex-mediated ER Ca^2+^ leakage in pathological conditions

### 4.1 SEC61 mutations and related pathologies

Several pathogenic mutations have been detected, as well in the genes coding for the proteins forming the Sec61 complex as in those coding for proteins associated with the complex (Lang et al., 2017; Sicking et al., 2021). These mutations result in various clinical phenotypes, including type 2 diabetes mellitus (T2D), immunodeficiency, tubulo-interstitial kidney disease and neutropenia (for mutations in SEC61A1). The occurrence of polycystic liver disease and colorectal cancer has been linked to SEC61B and that of glioblastoma multiforme, hepatocellular and renal cell carcinoma to SEC61C expression levels. Interestingly, mutations in proteins associated with the SEC61 complex recapitulate some of the above-mentioned diseases (T2D, polycystic liver disease) but can also lead to other diseases, such as the so-called congenital disorder of glycosylation (Lang et al., 2017; Sicking et al., 2021). Obviously, these mutations can affect each of the various aspects of Sec61 complex function and do not necessarily imply dysfunctional cellular Ca^2+^ handling. Interestingly, however, a number of those mutations were determined to impact ER Ca^2+^ leakage in mice (Schäuble et al., 2012) and humans (Schubert et al., 2018; Van Nieuwenhove et al., 2020) (Table 1).

**TABLE 1:** Identified SEC61A1 disease mutations and their effects on the ER Ca^2+^ leak. Description of SEC61A mutations leading to various diseases affecting kidney function, immunological behavior and/or metabolism, with special attention to the structural and functional consequences for the ER Ca^2+^ leak. TMD2 forms part of the lateral gate, while the pore ring is the name of the central constriction of Sec61α, which in its closed conformation is occupied by Loop 1, called the plug domain.

Mutation in SEC61A	Location mutated a.a	ER Ca^2+^ leak	Primary disease	Additional observations	References
V67G	Plug domain (Loop 1 between TMD1 and TMD2)	Increased	ADTKD	Also neutropenia	Van Nieuwenhove et al. (2020)
					Bolar et al. (2016)
V85D	Pore ring in TMD2	Increased	CVID		Schubert et al. (2018)
					Van Nieuwenhove et al. (2020)
Q92R	TMD2	Increased	SCN	Other leucocytes also affected but kidney function normal	Van Nieuwenhove et al. (2020)
T185A	TMD5 (very near to pore ring)	n.d	ADTKD		Bolar et al. (2016)
Y344H	Loop 7 (BiP-binding site)	Increased	Diabetes mellitus	Observed in mice	Schäuble et al. (2012)
					Lloyd et al. (2010)
E381*	Premature stop codon leading to haploinsufficiency	n.d	CVID		Schubert et al. (2018)

Abbreviations used: TMD, transmembrane domain; n.d., not determined; ADTKD, autosomal-dominant tubulo-interstitial kidney disease; CVID, common variable immunodeficiency; SCN, severe congenital neutropenia.

Common variable immunodeficiency (CVID) is a group of diseases of various origins that are generally characterized by impaired B-cell differentiation/function, resulting in low levels of antibody production and, consequently, recurrent infections. Two mutations in SEC61A1, a heterozygous missense mutation (V85D) and a nonsense mutation (E381*), have been linked to CVID (Schubert et al., 2018). SEC61A1-V85D, when expressed in HeLa cells, not only impaired protein translocation to the ER, but also resulted in a severe depletion of the ER Ca^2+^ store due to increased Ca^2+^ leakage out of the ER. SEC61A1-V85D expression selectively impaired the survival of plasma cells. This was due to the induction of unresolvable ER stress, leading to terminal UPR.

Autosomal dominant severe congenital neutropenia (SCN) forms another genetically heterogeneous group characterized by differentiation arrest in the formation of granulocytes. Mutations in over 20 genes are already involved in this pathology, while for many patients the responsible mutation has not yet been characterized. In a recent study, the point mutation Q92R in SEC61A1 was identified in a patient with severe congenital neutropenia (Van Nieuwenhove et al., 2020). Expression of SEC61A1-Q92R in HL-60 cells induced a 30% depletion of the ER Ca^2+^ store compared to wild-type SEC61A1. This was similar to the depletion observed when expressing in the same cells SEC61A1-V67G or SEC61A1-T185A, two heterozygous missense mutations already shown to result in autosomal-dominant tubulo-interstitial kidney disease (ADTKD) (Bolar et al., 2016). Additionally, SEC61A1-Q92R patient cells demonstrated an increased UPR and were more prone to apoptosis.

Interestingly, the ADTKD patients expressing the V67G mutation also displayed some neutropenia (Bolar et al., 2016). Similarly, the patient with the Q92R mutation was not only characterized by SCN, but also displayed B-cell maturation defects, reminiscent of the patients with SEC61A mutations leading to CVID, though harbored normal kidney function (Van Nieuwenhove et al., 2020).

Finally, investigation in a mouse model for T2D indicated that the missense mutation Y344H in loop 7 of murine Sec61α was linked to the occurrence in β cells of continuing ER stress leading to apoptosis and the subsequent development of T2D (Lloyd et al., 2010). The same mice also displayed hypercholesterolemia, hypertriglyceridemia, hepatomegaly, steatosis and, in older animals, hepatic cirrhosis. These observations are also relevant for humans, since the motif is fully conserved between mice and humans. Subsequent expression of Sec61α-Y344H in human HeLa cells led to increased ER Ca^2+^ leakage (Schäuble et al., 2012). Moreover, this mutation impairs binding of BiP to Sec61α, consistent with the observation that BiP limits ER Ca^2+^ leakage (see Section 3.4.1). Furthermore, these results strongly suggest that the gating of the Sec61 complex by BiP occurs via its binding to loop 7 of Sec61α. Interestingly, deletion of ERj6, one of the proteins assisting BiP in its regulation of Sec61 complex-mediated Ca^2+^ leakage, results in both mice (Ladiges et al., 2005) and humans (Synofzik et al., 2014) in pancreatic β cell failure and T2D, suggesting that increased Ca^2+^ leakage might form part of the mechanism involved.

It is presently not understood how the various SEC61A1 mutations form the origin of different clinical phenotypes, but this may be due to a combination of various functional effects, including but not limited to dysfunctional Ca^2+^ handling, and destabilization of the protein leading to lower expression levels. It is, however, clear that, with the exception of Y344H, which affects BiP binding, the other mutations linked to dysfunctional Ca^2+^ handling (V67G, V85D, Q92R, Y344H) are thus far all located in or near TMD2 (Table 1). V67G is located in the loop between TMD1 and TMD2 that forms a plug that seals and stabilizes the pore during the closed state (Linxweiler et al., 2013). V85D and Q92R, on the other hand, introduce a charge in the hydrophobic TMD2, which will likely affect the pore conformation and modify Ca^2+^-channel properties. Moreover, TMD2 and TMD7 form the so-called lateral gate that can allow/prevent lateral access to the central pore (Sicking et al., 2021).

In view of the structure of SEC61A, it may be expected that other mutations will be discovered that also affect its Ca^2+^ handling, which will help our understanding of the Ca^2+^-leak pathway.

### 4.2 Involvement of Sec61-mediated Ca^2+^ leakage in ER stress and UPR in disease

As detailed earlier, ER stress is due to either accumulation of unfolded or misfolded proteins in the ER lumen or due to Ca^2+^-store depletion. The UPR is an adaptive phenomenon aimed at reducing the unfolded protein burden (Hammadi et al., 2013; Hetz et al., 2020). A prolonged UPR is associated with ERAD. Unfolded proteins are retrotranslocated from the ER lumen to the cytoplasm, where they are degraded by the 26S proteasome (Wu and Rapoport, 2018). Prolonged ER stress will lead to apoptosis. Among all the signaling transduction pathways of ER stress and UPR, it is interesting here to focus on BiP overexpression. In the absence of ER stress, BiP maintains IRE1, PERK and ATF6 (Behnke et al., 2015; Pobre et al., 2019) in an inactive state. BiP also plugs the pore of the luminal side of the Sec61 complex (Hamman et al., 1997; Haigh and Johnson, 2002; Alder et al., 2005) and stabilizes the type 1 IP_3_R (Higo et al., 2010). During ER stress and UPR, a reorientation of BiP occurs: the chaperone binds to unfolded proteins and thus unhooks from the Sec61 complex, type 1 IP_3_R, IRE1, PERK and ATF6, concomitantly triggering Ca^2+^ leakage from the ER via the Sec61 complex (Figure 2). Very recently, using GCamP6, a genetically encoded Ca^2+^ indicator tethered to the ER membrane, evidence was presented that during the early phase of the UPR, the Sec61 complex evoked Ca^2+^ signals (Feliziani et al., 2022). Interestingly, these Ca^2+^ signals had physiological significance, as they contributed to PERK activation.

Ca^2+^ dysregulations as well as UPR perturbations have been associated with many diseases such as T2D (Özcan et al., 2006; Hotamisligil, 2010), cardiac pathologies (Minamino and Kitakaze, 2010; Hammadi et al., 2013; Luo et al., 2015) and cancer (Tsai and Weissman, 2010; Hammadi et al., 2013). Further questions arise now. Are ER stress and Ca^2+^ homeostasis independent mechanisms? What could be the link between these two mechanisms, and how could they be involved in these pathologies?

The involvement of the Sec61 complex in pathological contexts is beginning to be investigated. In the following sections, we will focus on cancer, T2D, cardiac ischemia-reperfusion and stunned myocardium with the Sec61 complex as a potential therapeutic target.

#### 4.2.1 Sec61 complex and cancer

Resistance to apoptosis is one of the major hallmarks of cancerous cells. New therapeutic strategies must focus on the induction of cell death. There is a direct link between ER Ca^2+^ homeostasis and cell survival. Any disruption of Ca^2+^ exchange between the ER and cytoplasm will have important consequences on the initiation of apoptosis. Cell death due to ER stress and a prolonged UPR is linked to ER Ca^2+^-store depletion (Zhang and Kaufman, 2008; Peters and Raghavan, 2011). In this context, the main route through which Ca^2+^ is released from the ER lumen during ER stress to switch the cell survival/apoptosis balance in favor of apoptosis is unknown. The Sec61 complex appears to be a good candidate to explain the mechanism of Ca^2+^-store depletion associated with ER stress and UPR.

The role of the Sec61 complex in cell death and ER stress has been investigated in the human cancerous cell line LNCaP (Hammadi et al., 2013). In this study, its involvement in ER stress as an ER Ca^2+^-leak channel was assessed using multiple ER stress/UPR inducers: brefeldin A, dithiothreitol and tunicamycin to trigger UPR as well as thapsigargin and puromycin to deplete ER Ca^2+^ stores in the absence of misfolded protein accumulation. In each condition, anisomycin both reduced ER stress and prevented depletion of the ER Ca^2+^ store, demonstrating that the Sec61 complex acts as a major ER Ca^2+^-leak channel during ER stress/UPR. Remarkably, inhibition of Sec61 complex-mediated Ca^2+^ leakage by anisomycin also inhibits apoptosis triggered by Ca^2+^ store depletion induced by thapsigargin and puromycin. As anisomycin can also activate a number of protein kinases, a puromycin/anisomycin pair (or equivalent) should always be used in these types of studies. Since the effect of one molecule blocks that of the other, their use allows us to rule out any non-specific action that would impede the correct interpretation of the results. The study by Hammadi *et al.* (Hammadi et al., 2013) therefore showed for the first time that the Sec61 complex controls ER Ca^2+^ content in stressed conditions. This finding indicates that pharmacological modulation of the Sec61 complex could promote cell survival.

Returning to the apoptosis resistance of cancer cells, BiP has been correlated with cancer malignancy (Virrey et al., 2008; Ni et al., 2011). Indeed, most cancerous cells overexpress BiP compared to normal cells. A study by Reddy *et al.* (Reddy et al., 2003) highlighted the link between BiP overexpression and drug resistance in cancer cells. Other studies clearly demonstrate that BiP is involved in the resistance of breast cancer cells to etoposide (an inhibitor of topoisomerase 2, used in chemotherapy) (Mandic et al., 2003) and to apoptosis (Fu et al., 2007). Moreover, BiP is overexpressed in ER stress, during UPR and in cancer cells (Ni et al., 2011; Farshbaf et al., 2020). On the one hand, BiP seals the luminal pore of the Sec61 complex (Hamman et al., 1998) and thus inhibits ER Ca^2+^ leakage (Schäuble et al., 2012; Hammadi et al., 2013). On the other hand, UPR and ER stress trigger BiP overexpression (Hammadi et al., 2013). Therefore, apoptosis resistance of cancerous cells could be partly due to a reduced ER Ca^2+^ leak at resting state involving the tandem Sec61 complex/BiP. Pharmacological modulation of the Ca^2+^ leak though the Sec61 complex could thus form a potential approach to force apoptosis-resistant cancer cells to undergo cell death and to enhance chemotherapy efficiency.

#### 4.2.2 Protection of human pancreatic islets from lipotoxicity by modulation of the Sec61 complex

T2D is associated with pancreatic β cell dysfunction and insulin resistance. This pathology is a major concern in health care worldwide. Its prevalence and incidence are rising, especially in developed countries such as western Europe (Khan et al., 2020). One of the major causes of T2D is a high-fat, high-sucrose diet. Indeed, excessive consumption of free fatty acids (FFAs) is correlated with a high risk for developing T2D (Risérus et al., 2009). In this context, palmitate is one of the main FFA in blood (Dey et al., 2007; Hommelberg et al., 2011; Leamy et al., 2014) that triggers deleterious effects called “lipotoxicity” (Bensellam et al., 2012). Palmitate-induced β cell lipotoxicity is associated with apoptosis involving numerous mechanisms, such as reactive oxygen species (ROS) production (Cnop, 2008; Fonseca et al., 2011), inflammation (Eguchi et al., 2012) and autophagy (Martino et al., 2012). Chronic exposure to FFA enhances ER stress/UPR in β cells (Özcan et al., 2006). In T2D, high insulin protein synthesis also triggers ER stress/UPR (Song et al., 2008; Åkerfeldt et al., 2008), as evidenced by ER stress marker overexpression (Back and Kaufman, 2012; Papa, 2012). Similarly, chemical chaperones used in a mouse model of T2D reduce ER stress and restore glucose homeostasis (Özcan et al., 2006). On the other hand, dysregulation of Ca^2+^ homeostasis is also associated with defective insulin release (Gwiazda et al., 2009). Interestingly, Cnop *et al.* (Cnop et al., 2014) analyzed the transcriptome of human islets after palmitate treatment. They demonstrated an enhanced level of SEC61α and BIP transcripts. Taken together, these data support the involvement of the Sec61 complex and Ca^2+^ leakage from the ER in palmitate-induced lipotoxicity in pancreatic β cells.

This hypothesis has been checked in the MIN6B1 cell line, obtained from a mouse insulinoma (Miyazaki et al., 1990; Ishihara et al., 1993). In these studies, the authors established and characterized a pancreatic β cell line that exhibits morphological and physiological characteristics (glucose metabolism and glucose-stimulated insulin secretion (GSIS)) similar to normal β cells. Physiologically more relevant, pharmacological modulation of the Sec61 complex was also performed in human pancreatic islets obtained from non-diabetic donors (Cassel et al., 2016). In this study, inhibition of the Sec61 complex with anisomycin prevented palmitate-induced ER Ca^2+^ depletion and reduced ER stress. It is well known that under physiological conditions, glucose stimulates insulin secretion by β cells. The GSIS assay is a method to investigate the physiological functionality of islets. One of the deleterious effects of palmitate-induced lipotoxicity is the decrease in insulin secretion in response to glucose. Using the GSIS method, it has been shown that the inhibition of the Sec61 complex by anisomycin restores glucose-induced insulin secretion in human islets treated with palmitate (Cassel et al., 2016).

In conclusion, Ca^2+^ leakage through the Sec61 complex appears to be a key element involved in T2D. One of the mechanisms by which palmitate causes lipotoxicity in pancreatic β cells occurs via ER stress associated with an increase in ER Ca^2+^-store depletion through the Sec61 complex. Therefore, pharmacological modulation of Sec61 opening could be a promising strategy for the treatment of ER stress-linked pathologies such as T2D (Figure 2).

#### 4.2.3 Cardioprotective role of the modulation of Sec61-mediated Ca^2+^ leakage during heart ischemia-reperfusion and cardiac infarcts

Cardiac infarction is one of the leading causes of mortality. Obstruction of a coronary artery induces ischemia in an area at risk, which is responsible for lesions due to apoptosis of cardiomyocytes. Reflow triggers further reperfusion injuries (Hausenloy et al., 2016). The role of ER Ca^2+^ handling in the regulation of heart rate, myocardial contraction, blood pressure and blood flow is the subject of considerable investigation. Ca^2+^ signaling is central for heart function, through its physiological role in excitation-contraction coupling as well as by its detrimental impact during heart failure and myocardial ischemia-reperfusion. During this latter condition, it is well accepted that the cytosolic accumulation of Ca^2+^ due to ER Ca^2+^ depletion via Ca^2+^-leak channels results in mitochondrial Ca^2+^ overload, which can trigger the opening of the mitochondrial permeability transition pore (mPTP), leading to cell death (Ovize et al., 2010; Al-Mawla et al., 2020; Ramachandra et al., 2020). During ischemia, the decrease in O_2_ uptake induces cellular acidosis, stimulating Na^+^/H^+^ exchange (for review, see Allen and Xiao, 2003). As a consequence, Na^+^ influx will be counterbalanced by the activity of the Na^+^/Ca^2+^ exchanger, further contributing to the [Ca^2+^]_cyt_ increase during ischemia. The initial Ca^2+^ release via ER Ca^2+^-leak channels is a crucial step in the cascade of events responsible for Ca^2+^ dysregulation and cell death. The dynamics and the amplitude of Ca^2+^ exchanged between subcellular compartments and especially between the ER and mitochondria are capital determinants of cell fate. Nevertheless, the overall signaling pathways are still not fully understood.

The role of the Sec61 complex in myocardial infarction has been investigated in mice (Al-Mawla et al., 2020). Mice were subjected to ischemia/reperfusion (I/R) protocols. In a complementary way, *in vitro* experiments were conducted on primary mouse cardiomyocytes in hypoxia/reperfusion (H/R) protocols, mimicking I/R at the cellular level. In these cells, the Sec61 complex was first shown to function as a Ca^2+^-leak channel. In cardiomyocytes, it is well known that during excitation-contraction coupling, type 2 RyRs are involved in Ca^2+^ release via a Ca^2+^-induced Ca^2+^ release mechanism. Interestingly, Sec61 complex activation mobilizes a RyR-independent Ca^2+^ pool that affects neither contraction nor RyR-dependent Ca^2+^ stores (Al-Mawla et al., 2020). These data are compatible with a compartmentalization of the Ca^2+^ stores: a puromycin-sensitive Ca^2+^ pool where Sec61 complexes are located (probably the ER) and a caffeine-sensitive Ca^2+^ pool containing RyR channels, located in the SR. Pharmacological pre-activation of the Sec61 complex with puromycin induces a preventive ER Ca^2+^ release from RyR-independent stores that consequently decreases the rate of Ca^2+^ increase in the cytosol as well as mitochondrial Ca^2+^ overload and mPTP opening during hypoxia. These data explain how puromycin, applied before H/R (pre-conditioning), significantly reduces cell death. *In vivo* cardioprotective experiments show that pharmacological modulation of the Sec61 complex (pre-conditioning) protects the mouse heart from I/R injury and reduces infarct size after I/R (Al-Mawla et al., 2020).

In conclusion, the Sec61 complex and its Ca^2+^ leak form a new paradigm in cardioprotection and in I/R injuries by functionally uncoupling Ca^2+^-dependent contraction from Ca^2+^-dependent cell fate.

#### 4.2.4 Pharmacological inhibition of the Sec61 complex improves contractile recovery in stunned myocardium

In the heart, ER stress and cytosolic Ca^2+^ overload also occur in myocardial stunning (Bolli and Marbán, 1999; Mariángelo et al., 2020). This contractile dysfunction occurs after brief episodes of ischemia with negative consequences on myocardium contraction despite the absence of cell death. Recently, an interesting article highlighted the beneficial effects of Sec61 complex inhibition (with emetine) in Ca^2+^ dysregulation and post-ischemic contractile dysfunction in stunned myocardium (Mariángelo et al., 2022). First, the authors show that inhibition of the Sec61 complex prior to I/R, prevents stunning-induced ER stress in rat hearts and improves post-ischemic mechanical recovery in stunned myocardium. In addition, Sec61 complex blockage reduced the I/R-induced increase in diastolic Ca^2+^ in mouse hearts.

Altogether, these data point out the capital role of Ca^2+^ leakage via the Sec61 complex in stunned myocardium. This work reinforces the potential therapeutic value of pharmacological modulation of Ca^2+^ leakage via the Sec61 complex to cope with the deleterious consequences of cardiac I/R.

## 5 Future perspectives and concluding remarks

From the above it thus appears that the Sec61 complex plays an important role in intracellular Ca^2+^ homeostasis beyond its central role in protein translocation through the ER membrane. This role in Ca^2+^ homeostasis is due to its functioning as a major ER Ca^2+^-leak channel. An uncontrolled Ca^2+^ leak from the ER to cytosol or mitochondria would be highly disruptive for normal cell behavior, and the Sec61 complex Ca^2+^-leak function is consequently controlled by various mechanisms. Channel closure is physiologically achieved by BiP from the luminal side (see Section 3.4.1) and by Ca^2+^/CaM from the cytosolic side (see Section 3.4.2). These regulatory mechanisms allow the Sec61 complex to modulate Ca^2+^ signaling, both in physiological and in patho (physio)logical settings.

As mentioned above, the ER Ca^2+^ content results from the dynamic equilibrium existing between Ca^2+^ release from the ER and its reuptake by SERCA pumps. The activity of both the Ca^2+^-release channels and SERCAs is finely regulated and depends to a large degree on [Ca^2+^]_cyt_ and [Ca^2+^]_ER_ (Berridge et al., 2003; Berridge, 2016), so that the ER Ca^2+^-store content is regulated in a dynamic way. Inhibition of the SERCA pumps by specific inhibitors (e.g., thapsigargin) incapacitates Ca^2+^ reuptake, uncovering the (normally compensated) activity of the ER Ca^2+^-leak channels. How does Ca^2+^ leakage via the Sec61 complex now fit in ER Ca^2+^ homeostasis? To answer this question, we can consider two scenarios.

In the first scenario, we assume in the cell a single, non-compartmentalized Ca^2+^ pool. This is, for example, the case in the human prostate cancer cell line LNCaP. In this cellular model, it has been shown that the acute activation of the Sec61 complex by puromycin induces partial emptying of a non-compartmentalized Ca^2+^ store. The rate of Ca^2+^ release is slower than that of IP_3_R- or RyR-mediated Ca^2+^ release (Van Coppenolle et al., 2004). After the puromycin response, the [Ca^2+^]_ER_ stabilizes to a lower value than at resting state. This value results from a new balance between Ca^2+^ leakage (via an undefined number of Sec61 complexes kept open by puromycin as well as by other types of ER Ca^2+^-leak channels that are presumably present) and SERCA reuptake activity. One element to consider is that during the acute action of puromycin (or emetine), only the Sec61 complexes involved in translation are sensitive to puromycin. Complexes that do not participate in translation remain impermeable to Ca^2+^. During chronic puromycin or emetine perfusion, their concentration should therefore be chosen so that it does modulate the ER Ca^2+^ levels without any effect on translation (Hammadi et al., 2013). Beyond that, the cytotoxic effects of these antibiotics will take over. Thus, contrary to what we might initially consider given the pore diameter of the Sec61 complex, puromycin, at the concentration used, whether acute or chronic, does not cause a total emptying of the ER Ca^2+^ stores. This does not exclude the possibility that other modulatory mechanisms can also act on the Sec61 complex during long-term perfusion. This point requires further investigation. Subsequently, the residual ER Ca^2+^-store content can be mobilized following IP_3_R or RyR activation, thereby generating a new Ca^2+^ steady-state at an even lower value (Van Coppenolle et al., 2004). At this point, it is important to note that the activation of the Ca^2+^-leak channels, the IP_3_Rs, the RyRs or the inhibition of SERCAs do not manage to completely drain the ER. Complete release of ER Ca^2+^ will only be possible with Ca^2+^ ionophores, e.g., ionomycin.

A second scenario takes into account the compartmentalization of the Ca^2+^ store in an ER and an SR Ca^2+^ pool, which can function in an either partially or totally independent way. This case was discussed in primary mouse cardiomyocytes (Al-Mawla et al., 2020) in Section 4.2.3. The modes of action of puromycin and emetine will be similar to those described in the previous paragraph. However, in such a cellular model, there is a functional dichotomy between ER and SR. Thus, the action of emetine, acting on the Sec61 complex of the ER, would have no significant impact on the caffeine-sensitive pool of the SR, expressing RyRs and thus controlling excitation-contraction coupling.

ER stress has a complex relationship with intracellular Ca^2+^ handling (Kiviluoto et al., 2013; Carreras-Sureda et al., 2018; Groenendyk et al., 2021). On the one hand, one of the consequences of unalleviated ER stress is that it leads to increased Ca^2+^ release from the ER, though on the other hand, ER Ca^2+^-store depletion is also a trigger for ER stress. Independent of its origin, in ER stress conditions, BiP will detach from its various physiological binding partners to help alleviate ER stress by binding to unfolded or misfolded proteins as well as to allow the UPR to start. Consequently, the regulation of ER Ca^2+^ leakage through the Sec61 complex is abrogated as BiP dissociates, and Ca^2+^ leakage increases. This increase in Ca^2+^ leakage is a factor that can lead to cell death. As we have highlighted above (see Section 4.2), inhibition of Sec61 Ca^2+^ leakage can antagonize these effects and lead to cell survival (Figure 2).

As Sec61 complex-mediated Ca^2+^ leakage appears dysregulated in various pathological situations, we pose that Sec61 forms a valid therapeutic target and that its modulation, activation in cancer (see section 4.2.1) and inhibition in T2D (see section 4.2.2) can lead to beneficial effects. Although more complex, Sec61 complex modulation can also have a role in cardioprotection (see section 4.2.3 and section 4.2.4).

From all these studies, what therapeutic conclusions, based on pharmacological modulation of Ca^2+^ leakage via the Sec61 complex, can be inferred? First, it is necessary to determine, on a case-by-case basis, the concentration and time of action of molecules of interest that do not significantly alter protein translation while acting on Ca^2+^ permeability. As ER Ca^2+^ leakage modulates the cellular death/survival balance, we must therefore consider the intended objective of the treatments. In cancer, it will be to promote cell death, but in other cases (e.g., during ischemic stress (heart, brain, kidney) or gluco-lipotoxicity (T2D)), it will be to promote cell survival. In the case of cancer, it would therefore likely be necessary to use Ca^2+^-leakage inducers, which could be used in combination with existing therapies to act preferentially on cancer cells rather than on healthy cells. We assume that a small dose of these molecules could already tip the scales towards cell death. In the case of T2D or stunned myocardium, however, it is likely that longer-term treatments would be more effective. Conversely, in the framework of cardiac I/R in a mouse model, a puromycin bolus has already shown its efficacy, both *in vivo* and *in vitro*.

Moreover, we anticipate that future research will uncover the role of Sec61 complex-mediated Ca^2+^ leakage in additional pathological situations. Since the basis of neurodegenerative diseases lies in the accumulation of unfolded proteins and Ca^2+^ dysregulation, neuroprotective strategies involving the regulation of Ca^2+^-leak channels in general and the Sec61 complex in particular will probably emerge. Obvious possible candidates include neurodegenerative diseases such as Parkinson’s disease, Alzheimer’s disease, Huntington’s disease, and Creutzfeld-Jacob disease. The role of intracellular Ca^2+^ in general and of ER Ca^2+^ in particular (Ureshino et al., 2019; da Costa et al., 2020; Lim et al., 2021; Callens et al., 2021; Kovacs et al., 2021; Guan et al., 2021; Huang et al., 2022; Ge et al., 2022; Kim et al., 2022) as well as of ER stress and accumulation of misfolded proteins (da Costa et al., 2020; Guan et al., 2021; Kim et al., 2022; Ren et al., 2021; Yasmeen et al., 2022; Shacham et al., 2019; Chakraborty et al., 2005) have indeed already been demonstrated in several neurodegenerative diseases.

Expanding from T2D, we can also expect a role for Sec61 complex-mediated Ca^2+^ leakage in other diseases in which a relation with ER stress and Ca^2+^ signaling was demonstrated, such as non-alcoholic fatty liver disease (Lebeaupin et al., 2018; Chen et al., 2021).

A separate avenue for future research constitutes lysosomal diseases. Indeed, the ER and lysosomes are in close connection and at least small Ca^2+^ signals originating from IP_3_R can feed into lysosomes (Atakpa et al., 2018). It is therefore possible that Ca^2+^ ions leaking from the ER by the Sec61 complex can similarly impact lysosomal Ca^2+^. This understanding could be relevant for various lysosomal storage diseases, including Niemann-Pick type C disease and Gaucher disease, in which ER and/or lysosomal Ca^2+^ appears dysregulated (Lloyd-Evans and Platt, 2011; Liu and Lieberman, 2019).

Finally, until now, only a limited number of mutations in Sec61 genes and in other components of the complex have been described (see section 4.1). We therefore anticipate that future work will uncover additional (human) mutations, which will shed new light on the mechanism of action, regulation and physiological and pathological roles of the Sec61 complex in Ca^2+^ homeostasis.

Taken together, these findings strengthen the evidence that Sec61 complex-mediated Ca^2+^ leakage plays an important role in intracellular Ca^2+^ homeostasis, is involved in major pathologies when performing in an unregulated way, and thus forms a promising therapeutic target. Further research on the topic will therefore be of great importance to fully elucidate its physiological as well as its patho (physio)logical role.

## References

[B1] Åkerfeldt M. C. Howes J. Chan J. Y. Stevens V. A. Boubenna N. McGuire H. M. (2008). Cytokine-induced β-cell death is independent of endoplasmic reticulum stress signaling. Diabetes 57 (11), 3034–3044. 10.2337/db07-1802 18591394 PMC2570400

[B2] Al-Mawla R. Ducrozet M. Tessier N. Païta L. Pillot B. Gouriou Y. (2020). Acute induction of translocon-mediated Ca^2+^ leak protects cardiomyocytes against ischemia/reperfusion injury. Cells 9 (5), 1319. 10.3390/cells9051319 PMC7290748 32466308

[B3] Alder N. N. Shen Y. Brodsky J. L. Hendershot L. M. Johnson A. E. (2005). The molecular mechanisms underlying BiP-mediated gating of the Sec61 translocon of the endoplasmic reticulum. J. Cell Biol. 168 (3), 389–399. 10.1083/jcb.200409174 15684029 PMC2171714

[B4] Allen D. G. Xiao X. H. (2003). Role of the cardiac Na^+^/H^+^ exchanger during ischemia and reperfusion. Cardiovasc. Res. 57 (4), 934–941. 10.1016/s0008-6363(02)00836-2 12650871

[B5] Annaert W. G. Levesque L. Craessaerts K. Dierinck I. Snellings G. Westaway D. (1999). Presenilin 1 controls γ-secretase processing of amyloid precursor protein in pre-Golgi compartments of hippocampal neurons. J. Cell Biol. 147 (2), 277–294. 10.1083/jcb.147.2.277 10525535 PMC2174229

[B6] Atakpa P. Thillaiappan N. B. Mataragka S. Prole D. L. Taylor C. W. (2018). IP_3_ receptors preferentially associate with ER-lysosome contact sites and selectively deliver Ca^2+^ to lysosomes. Cell Rep. 25 (11), 3180–3193. e7. 10.1016/j.celrep.2018.11.064 30540949 PMC6302550

[B7] Back S. H. Kaufman R. J. (2012). Endoplasmic reticulum stress and type 2 diabetes. Annu. Rev. Biochem. 81, 767–793. 10.1146/annurev-biochem-072909-095555 22443930 PMC3684428

[B8] Bagur R. Hajnóczky G. (2017). Intracellular Ca^2+^ sensing: Its role in calcium homeostasis and signaling. Mol. Cell 66 (6), 780–788. 10.1016/j.molcel.2017.05.028 28622523 PMC5657234

[B9] Bai L. Wang T. Zhao G. Kovach A. Li H. (2018). The atomic structure of a eukaryotic oligosaccharyl transferase complex. Nature 555 (7696), 328–333. 10.1038/nature25755 29466327 PMC6112861

[B10] Bandara S. Malmersjö S. Meyer T. (2013). Regulators of calcium homeostasis identified by inference of kinetic model parameters from live single cells perturbed by siRNA. Sci. Signal. 6 (283), ra56. 10.1126/scisignal.2003649 23838183 PMC3897207

[B11] Baron L. Paatero A. O. Morel J. D. Impens F. Guenin-Macé L. Saint-Auret S. (2016). Mycolactone subverts immunity by selectively blocking the Sec61 translocon. J. Exp. Med. 213 (13), 2885–2896. 10.1084/jem.20160662 27821549 PMC5154940

[B12] Becker T. Bhushan S. Jarasch A. Armache J. P. Funes S. Jossinet F. (2009). Structure of monomeric yeast and mammalian Sec61 complexes interacting with the translating ribosome. Science 326 (5958), 1369–1373. 10.1126/science.1178535 19933108 PMC2920595

[B13] Behnke J. Feige M. J. Hendershot L. M. (2015). BiP and its nucleotide exchange factors Grp170 and Sil1: Mechanisms of action and biological functions. J. Mol. Biol. 427 (7), 1589–1608. 10.1016/j.jmb.2015.02.011 25698114 PMC4356644

[B14] Bensellam M. Laybutt D. R. Jonas J. C. (2012). The molecular mechanisms of pancreatic β-cell glucotoxicity: Recent findings and future research directions. Mol. Cell. Endocrinol. 364 (1), 1–27. 10.1016/j.mce.2012.08.003 22885162

[B15] Berg B. Clemons W. M. Collinson I. Modis Y. Hartmann E. Harrison S. C. (2004). X-ray structure of a protein-conducting channel. Nature 427 (6969), 36–44. 10.1038/nature02218 14661030

[B16] Berridge M. J. Bootman M. D. Roderick H. L. (2003). Calcium signalling: Dynamics, homeostasis and remodelling. Nat. Rev. Mol. Cell Biol. 4 (7), 517–529. 10.1038/nrm1155 12838335

[B17] Berridge M. J. (2006). Calcium microdomains: Organization and function. Cell Calcium 40 (5-6), 405–412. 10.1016/j.ceca.2006.09.002 17030366

[B18] Berridge M. J. Lipp P. Bootman M. D. (2000). The versatility and universality of calcium signalling. Nat. Rev. Mol. Cell Biol. 1 (1), 11–21. 10.1038/35036035 11413485

[B19] Berridge M. J. (2002). The endoplasmic reticulum: A multifunctional signaling organelle. Cell Calcium 32 (5), 235–249. 10.1016/s0143416002001823 12543086

[B20] Berridge M. J. (2016). The inositol trisphosphate/calcium signaling pathway in health and disease. Physiol. Rev. 96 (4), 1261–1296. 10.1152/physrev.00006.2016 27512009

[B21] Bhadra P. Dos Santos S. Gamayun I. Pick T. Neumann C. Ogbechi J. (2021). Mycolactone enhances the Ca^2+^ leak from endoplasmic reticulum by trapping Sec61 translocons in a Ca^2+^ permeable state. Biochem. J. 478 (22), 4005–4024. 10.1042/BCJ20210345 34726690 PMC8650850

[B22] Blau M. Mullapudi S. Becker T. Dudek J. Zimmermann R. Penczek P. A. (2005). ERj1p uses a universal ribosomal adaptor site to coordinate the 80S ribosome at the membrane. Nat. Struct. Mol. Biol. 12 (11), 1015–1016. 10.1038/nsmb998 16244660

[B23] Bolar N. A. Golzio C. Živná M. Hayot G. Van Hemelrijk C. Schepers D. (2016). Heterozygous loss-of-function SEC61A1 mutations cause autosomal-dominant tubulo-interstitial and glomerulocystic kidney disease with anemia. Am. J. Hum. Genet. 99 (1), 174–187. 10.1016/j.ajhg.2016.05.028 27392076 PMC5005467

[B24] Bolli R. Marbán E. (1999). Molecular and cellular mechanisms of myocardial stunning. Physiol. Rev. 79 (2), 609–634. 10.1152/physrev.1999.79.2.609 10221990

[B25] Bootman M. D. Bultynck G. (2020). Fundamentals of cellular calcium signaling: A primer. Cold Spring Harb. Perspect. Biol. 12 (1), a038802. 10.1101/cshperspect.a038802 31427372 PMC6942118

[B26] Braunger K. Pfeffer S. Shrimal S. Gilmore R. Berninghausen O. Mandon E. C. (2018). Structural basis for coupling protein transport and N-glycosylation at the mammalian endoplasmic reticulum. Science 360 (6385), 215–219. 10.1126/science.aar7899 29519914 PMC6319373

[B27] Callens M. Kraskovskaya N. Derevtsova K. Annaert W. Bultynck G. Bezprozvanny I. (2021). The role of Bcl-2 proteins in modulating neuronal Ca^2+^ signaling in health and in Alzheimer’s disease. Biochim. Biophys. Acta. Mol. Cell Res. 1868 (6), 118997. 10.1016/j.bbamcr.2021.118997 33711363 PMC8041352

[B28] Camello C. Lomax R. Petersen O. H. Tepikin A. V. (2002). Calcium leak from intracellular stores—The enigma of calcium signalling. Cell Calcium 32 (5), 355–361. 10.1016/s0143416002001926 12543095

[B29] Carreras-Sureda A. Pihán P. Hetz C. (2018). Calcium signaling at the endoplasmic reticulum: Fine-tuning stress responses. Cell Calcium 70, 24–31. 10.1016/j.ceca.2017.08.004 29054537

[B30] Cassel R. Ducreux S. Alam M. R. Dingreville F. Berlé C. Burda-Jacob K. (2016). Protection of human pancreatic islets from lipotoxicity by modulation of the translocon. PLoS One 11 (2), e0148686. 10.1371/journal.pone.0148686 26862742 PMC4749224

[B31] Chakraborty C. Nandi S. Jana S. (2005). Prion disease: A deadly disease for protein misfolding. Curr. Pharm. Biotechnol. 6 (2), 167–177. 10.2174/1389201053642321 15853695

[B32] Chami M. Oulès B. Szabadkai G. Tacine R. Rizzuto R. Paterlini-Bréchot P. (2008). Role of SERCA1 truncated isoform in the proapoptotic calcium transfer from ER to mitochondria during ER stress. Mol. Cell 32 (5), 641–651. 10.1016/j.molcel.2008.11.014 19061639 PMC2676567

[B33] Chen C. C. Hsu L. W. Chen K. D. Chiu K. W. Chen C. L. Huang K. T. (2021). Emerging roles of calcium signaling in the development of non-alcoholic fatty liver disease. Int. J. Mol. Sci. 23 (1), 256. 10.3390/ijms23010256 35008682 PMC8745268

[B34] Chen W. Helenius J. Braakman I. Helenius A. (1995). Cotranslational folding and calnexin binding during glycoprotein synthesis. Proc. Natl. Acad. Sci. U. S. A. 92 (14), 6229–6233. 10.1073/pnas.92.14.6229 7541532 PMC41491

[B35] Cheng H. Lederer W. J. (2008). Calcium sparks. Physiol. Rev. 88 (4), 1491–1545. 10.1152/physrev.00030.2007 18923188

[B36] Chevet E. Wong H. N. Gerber D. Cochet C. Fazel A. Cameron P. H. (1999). Phosphorylation by CK2 and MAPK enhances calnexin association with ribosomes. EMBO J. 18 (13), 3655–3666. 10.1093/emboj/18.13.3655 10393181 PMC1171443

[B37] Chin D. Means A. R. (2000). Calmodulin: A prototypical calcium sensor. Trends Cell Biol. 10 (8), 322–328. 10.1016/s0962-8924(00)01800-6 10884684

[B38] Cnop M. Abdulkarim B. Bottu G. Cunha D. A. Igoillo-Esteve M. Masini M. (2014). RNA sequencing identifies dysregulation of the human pancreatic islet transcriptome by the saturated fatty acid palmitate. Diabetes 63 (6), 1978–1993. 10.2337/db13-1383 24379348

[B39] Cnop M. (2008). Fatty acids and glucolipotoxicity in the pathogenesis of Type 2 diabetes. Biochem. Soc. Trans. 36 (3), 348–352. 10.1042/BST0360348 18481955

[B40] Conti B. J. Devaraneni P. K. Yang Z. David L. L. Skach W. R. (2015). Cotranslational stabilization of Sec62/63 within the ER Sec61 translocon is controlled by distinct substrate-driven translocation events. Mol. Cell 58 (2), 269–283. 10.1016/j.molcel.2015.02.018 25801167 PMC4402133

[B41] Cross B. C. S. McKibbin C. Callan A. C. Roboti P. Piacenti M. Rabu C. (2009). Eeyarestatin I inhibits Sec61-mediated protein translocation at the endoplasmic reticulum. J. Cell Sci. 122 (23), 4393–4400. 10.1242/jcs.054494 19903691 PMC2779136

[B42] Crowley K. S. Liao S. Worrell V. E. Reinhart G. D. Johnson A. E. (1994). Secretory proteins move through the endoplasmic reticulum membrane via an aqueous, gated pore. Cell 78 (3), 461–471. 10.1016/0092-8674(94)90424-3 8062388

[B43] da Costa C. A. Manaa W. E. Duplan E. Checler F. (2020). The endoplasmic reticulum stress/unfolded protein response and their contributions to Parkinson’s disease physiopathology. Cells 9 (11), 2495. 10.3390/cells9112495 PMC7698446 33212954

[B44] Daimon M. Susa S. Suzuki K. Kato T. Yamatani K. Sasaki H. (1997). Identification of a human cDNA homologue to the Drosophila translocation protein 1 (Dtrp1). Biochem. Biophys. Res. Commun. 230 (1), 100–104. 10.1006/bbrc.1996.5892 9020021

[B45] Danese A. Leo S. Rimessi A. Wieckowski M. R. Fiorica F. Giorgi C. (2021). Cell death as a result of calcium signaling modulation: A cancer-centric prospective. Biochim. Biophys. Acta. Mol. Cell Res. 1868 (8), 119061. 10.1016/j.bbamcr.2021.119061 33991539

[B46] De Strooper B. Saftig P. Craessaerts K. Vanderstichele H. Guhde G. Annaert W. (1998). Deficiency of presenilin-1 inhibits the normal cleavage of amyloid precursor protein. Nature 391 (6665), 387–390. 10.1038/34910 9450754

[B47] Dejgaard K. Theberge J. F. Heath-Engel H. Chevet E. Tremblay M. L. Thomas D. Y. (2010). Organization of the Sec61 translocon, studied by high resolution native electrophoresis. J. Proteome Res. 9 (4), 1763–1771. 10.1021/pr900900x 20112977

[B48] Demangel C. High S. (2018). Sec61 blockade by mycolactone: A central mechanism in Buruli ulcer disease. Biol. Cell 110 (11), 237–248. 10.1111/boc.201800030 30055020

[B49] Demangel C. (2021). Immunity against Mycobacterium ulcerans - the subversive role of mycolactone. Immunol. Rev. 301 (1), 209–221. 10.1111/imr.12956 33607704

[B50] Demangel C. Stinear T. P. Cole S. T. (2009). Buruli ulcer: Reductive evolution enhances pathogenicity of Mycobacterium ulcerans. Nat. Rev. Microbiol. 7 (1), 50–60. 10.1038/nrmicro2077 19079352

[B51] Dey D. Pal B. C. Biswas T. Roy S. S. Bandyopadhyay A. Mandal S. K. (2007). A lupinoside prevented fatty acid induced inhibition of insulin sensitivity in 3T3 L1 adipocytes. Mol. Cell. Biochem. 300 (1), 149–157. 10.1007/s11010-006-9378-1 17149545

[B52] Dudek J. Greiner M. Müller A. Hendershot L. M. Kopsch K. Nastainczyk W. (2005). ERj1p has a basic role in protein biogenesis at the endoplasmic reticulum. Nat. Struct. Mol. Biol. 12 (11), 1008–1014. 10.1038/nsmb1007 16244664

[B53] Egea P. F. Stroud R. M. (2010). Lateral opening of a translocon upon entry of protein suggests the mechanism of insertion into membranes. Proc. Natl. Acad. Sci. U. S. A. 107 (40), 17182–17187. 10.1073/pnas.1012556107 20855604 PMC2951439

[B54] Egea P. F. Stroud R. M. Walter P. (2005). Targeting proteins to membranes: Structure of the signal recognition particle. Curr. Opin. Struct. Biol. 15 (2), 213–220. 10.1016/j.sbi.2005.03.007 15837181

[B55] Eguchi K. Manabe I. Oishi-Tanaka Y. Ohsugi M. Kono N. Ogata F. (2012). Saturated fatty acid and TLR signaling link β cell dysfunction and islet inflammation. Cell Metab. 15 (4), 518–533. 10.1016/j.cmet.2012.01.023 22465073

[B56] Eida A. A. Mahmud T. (2019). The secondary metabolite pactamycin with potential for pharmaceutical applications: Biosynthesis and regulation. Appl. Microbiol. Biotechnol. 103 (11), 4337–4345. 10.1007/s00253-019-09831-x 31025074 PMC6545121

[B57] En J. Goto M. Nakanaga K. Higashi M. Ishii N. Saito H. (2008). Mycolactone is responsible for the painlessness of Mycobacterium ulcerans infection (Buruli ulcer) in a murine Study. Infect. Immun. 76 (5), 2002–2007. 10.1128/IAI.01588-07 18316387 PMC2346717

[B58] Erdmann F. Schäuble N. Lang S. Jung M. Honigmann A. Ahmad M. (2011). Interaction of calmodulin with Sec61α limits Ca^2+^ leakage from the endoplasmic reticulum. EMBO J. 30 (1), 17–31. 10.1038/emboj.2010.284 21102557 PMC3020109

[B59] Farshbaf M. Khosroushahi A. Y. Mojarad-Jabali S. Zarebkohan A. Valizadeh H. Walker P. R. (2020). Cell surface GRP78: An emerging imaging marker and therapeutic target for cancer. J. Control. Release 328, 932–941. 10.1016/j.jconrel.2020.10.055 33129921

[B60] Feliziani C. Fernandez M. Quassollo G. Holstein D. Bairo S. M. Paton J. C. (2022). Ca^2+^ signalling system initiated by endoplasmic reticulum stress stimulates PERK activation. Cell Calcium 106, 102622. 10.1016/j.ceca.2022.102622 35908318 PMC9982837

[B61] Flourakis M. Van Coppenolle F. Lehen’kyi V. Beck B. Skryma R. Prevarskaya N. (2006). Passive calcium leak via translocon is a first step for iPLA2-pathway regulated store operated channels activation. FASEB J. 20 (8), 1215–1217. 10.1096/fj.05-5254fje 16611832

[B62] Fonseca S. G. Gromada J. Urano F. (2011). Endoplasmic reticulum stress and pancreatic β cell death. Trends Endocrinol. Metab. 22 (7), 266–274. 10.1016/j.tem.2011.02.008 21458293 PMC3130122

[B63] Foskett J. K. White C. Cheung K. H. Mak D. O. D. (2007). Inositol trisphosphate receptor Ca^2+^ release channels. Physiol. Rev. 87 (2), 593–658. 10.1152/physrev.00035.2006 17429043 PMC2901638

[B64] Fu Y. Li J. Lee A. S. (2007). GRP78/BiP inhibits endoplasmic reticulum BIK and protects human breast cancer cells against estrogen starvation–induced apoptosis. Cancer Res. 67 (8), 3734–3740. 10.1158/0008-5472.CAN-06-4594 17440086

[B65] Gallegos-Gómez M. L. Greotti E. López-Méndez M. C. Sánchez-Vázquez V. H. Arias J. M. Guerrero-Hernández A. (2018). The trans Golgi region is a labile intracellular Ca^2+^ store sensitive to emetine. Sci. Rep. 8, 17143. 10.1038/s41598-018-35280-z 30464185 PMC6249204

[B66] Gamayun I. O’Keefe S. Pick T. Klein M. C. Nguyen D. McKibbin C. (2019). Eeyarestatin compounds selectively enhance Sec61-mediated Ca^2+^ leakage from the endoplasmic reticulum. Cell Chem. Biol. 26 (4), 571–583. e6. 10.1016/j.chembiol.2019.01.010 30799222 PMC6483976

[B67] Ge M. Zhang J. Chen S. Huang Y. Chen W. He L. (2022). Role of calcium homeostasis in Alzheimer’s disease. Neuropsychiatr. Dis. Treat. 18, 487–498. 10.2147/NDT.S350939 35264851 PMC8901263

[B68] Gemmer M. Förster F. (2020). A clearer picture of the ER translocon complex. J. Cell Sci. 133 (3), jcs231340. 10.1242/jcs.231340 32019826

[B69] Gérard S. F. Hall B. S. Zaki A. M. Corfield K. A. Mayerhofer P. U. Costa C. (2020). Structure of the inhibited state of the Sec translocon. Mol. Cell 79 (3), 406–415. e7. 10.1016/j.molcel.2020.06.013 32692975 PMC7427319

[B70] Giorgi C. Danese A. Missiroli S. Patergnani S. Pinton P. (2018). Calcium dynamics as a machine for decoding signals. Trends Cell Biol. 28 (4), 258–273. 10.1016/j.tcb.2018.01.002 29409699

[B71] Giunti R. Gamberucci A. Fulceri R. Bánhegyi G. Benedetti A. (2007). Both translocon and a cation channel are involved in the passive Ca^2+^ leak from the endoplasmic reticulum: A mechanistic study on rat liver microsomes. Arch. Biochem. Biophys. 462 (1), 115–121. 10.1016/j.abb.2007.03.039 17481572

[B72] Gogala M. Becker T. Beatrix B. Armache J. P. Barrio-Garcia C. Berninghausen O. (2014). Structures of the Sec61 complex engaged in nascent peptide translocation or membrane insertion. Nature 506 (7486), 107–110. 10.1038/nature12950 24499919

[B73] Greiner M. Kreutzer B. Lang S. Jung V. Cavalié A. Unteregger G. (2011). Sec62 protein level is crucial for the ER stress tolerance of prostate cancer. Prostate 71 (10), 1074–1083. 10.1002/pros.21324 21557272

[B74] Groenendyk J. Agellon L. B. Michalak M. (2021). Calcium signaling and endoplasmic reticulum stress. Int. Rev. Cell Mol. Biol. 363, 1–20. 10.1016/bs.ircmb.2021.03.003 34392927

[B75] Grotzke J. E. Kozik P. Morel J. D. Impens F. Pietrosemoli N. Cresswell P. (2017). Sec61 blockade by mycolactone inhibits antigen cross-presentation independently of endosome-to-cytosol export. Proc. Natl. Acad. Sci. U. S. A. 114 (29), E5910–E5919. 10.1073/pnas.1705242114 28679634 PMC5530691

[B76] Grudnik P. Bange G. Sinning I. (2009). Protein targeting by the signal recognition particle. Biol. Chem. 390 (8), 775–782. 10.1515/BC.2009.102 19558326

[B77] Guan P. P. Cao L. L. Yang Y. Wang P. (2021). Calcium ions aggravate Alzheimer’s disease through the aberrant activation of neuronal networks, leading to synaptic and cognitive deficits. Front. Mol. Neurosci. 14, 757515. 10.3389/fnmol.2021.757515 34924952 PMC8674839

[B78] Gwiazda K. S. Yang T. L. B. Lin Y. Johnson J. D. (2009). Effects of palmitate on ER and cytosolic Ca^2+^ homeostasis in β-cells. Am. J. Physiol. Endocrinol. Metab. 296 (4), E690–E701. 10.1152/ajpendo.90525.2008 19141690

[B79] Haigh N. G. Johnson A. E. (2002). A new role for BiP: Closing the aqueous translocon pore during protein integration into the ER membrane. J. Cell Biol. 156 (2), 261–270. 10.1083/jcb.200110074 11807091 PMC2199230

[B80] Hall B. S. Hill K. McKenna M. Ogbechi J. High S. Willis A. E. (2014). The pathogenic mechanism of the Mycobacterium ulcerans virulence factor, mycolactone, depends on blockade of protein translocation into the ER. PLoS Pathog. 10 (4), e1004061. 10.1371/journal.ppat.1004061 24699819 PMC3974873

[B81] Hamada K. Mikoshiba K. (2020). IP3 receptor plasticity underlying diverse functions. Annu. Rev. Physiol. 82, 151–176. 10.1146/annurev-physiol-021119-034433 31730387

[B82] Hammadi M. Oulidi A. Gackière F. Katsogiannou M. Slomianny C. Roudbaraki M. (2013). Modulation of ER stress and apoptosis by endoplasmic reticulum calcium leak via translocon during unfolded protein response: Involvement of GRP78. FASEB J. 27 (4), 1600–1609. 10.1096/fj.12-218875 23322163

[B83] Hamman B. D. Chen J. C. Johnson E. E. Johnson A. E. (1997). The aqueous pore through the translocon has a diameter of 40–60 Å during cotranslational protein translocation at the ER membrane. Cell 89 (4), 535–544. 10.1016/s0092-8674(00)80235-4 9160745

[B84] Hamman B. D. Hendershot L. M. Johnson A. E. (1998). BiP maintains the permeability barrier of the ER membrane by sealing the lumenal end of the translocon pore before and early in translocation. Cell 92 (6), 747–758. 10.1016/s0092-8674(00)81403-8 9529251

[B85] Hausenloy D. J. Barrabes J. A. Bøtker H. E. Davidson S. M. Di Lisa F. Downey J. (2016). Ischaemic conditioning and targeting reperfusion injury: A 30 year voyage of discovery. Basic Res. Cardiol. 111 (6), 70. 10.1007/s00395-016-0588-8 27766474 PMC5073120

[B86] Haßdenteufel S. Johnson N. Paton A. W. Paton J. C. High S. Zimmermann R. (2018). Chaperone-mediated Sec61 channel gating during ER import of small precursor proteins overcomes Sec61 inhibitor-reinforced energy barrier. Cell Rep. 23 (5), 1373–1386. 10.1016/j.celrep.2018.03.122 29719251 PMC5946456

[B87] Heritage D. Wonderlin W. F. (2001). Translocon pores in the endoplasmic reticulum are permeable to a neutral, polar molecule. J. Biol. Chem. 276 (25), 22655–22662. 10.1074/jbc.M102409200 11303028

[B88] Hetz C. Zhang K. Kaufman R. J. (2020). Mechanisms, regulation and functions of the unfolded protein response. Nat. Rev. Mol. Cell Biol. 21 (8), 421–438. 10.1038/s41580-020-0250-z 32457508 PMC8867924

[B89] Higo T. Hamada K. Hisatsune C. Nukina N. Hashikawa T. Hattori M. (2010). Mechanism of ER stress-induced brain damage by IP_3_ Receptor. Neuron 68 (5), 865–878. 10.1016/j.neuron.2010.11.010 21145001

[B90] Hommelberg P. P. H. Plat J. Sparks L. M. Schols A. M. W. J. van Essen A. L. M. Kelders M. C. J. M. (2011). Palmitate-induced skeletal muscle insulin resistance does not require NF-κB activation. Cell. Mol. Life Sci. 68 (7), 1215–1225. 10.1007/s00018-010-0515-3 20820848 PMC3056136

[B91] Hotamisligil G. S. (2010). Endoplasmic reticulum stress and the inflammatory basis of metabolic disease. Cell 140 (6), 900–917. 10.1016/j.cell.2010.02.034 20303879 PMC2887297

[B92] Huang D. X. Yu X. Yu W. J. Zhang X. M. Liu C. Liu H. P. (2022). Calcium signaling regulated by cellular membrane systems and calcium homeostasis perturbed in Alzheimer’s disease. Front. Cell Dev. Biol. 10, 834962. 10.3389/fcell.2022.834962 35281104 PMC8913592

[B93] Hummel H. Böck A. Bock A. (1987). 23S ribosomal RNA mutations in halobacteria conferring resistance to the anti-80S ribosome targeted antibiotic anisomycin. Nucleic Acids Res. 15 (6), 2431–2443. 10.1093/nar/15.6.2431 3562233 PMC340661

[B94] Hwang J. Qi L. (2018). Quality control in the endoplasmic reticulum: Crosstalk between ERAD and UPR pathways. Trends biochem. Sci. 43 (8), 593–605. 10.1016/j.tibs.2018.06.005 30056836 PMC6327314

[B95] Ichhaporia V. P. Hendershot L. M. (2021). Role of the HSP70 co-chaperone SIL1 in health and disease. Int. J. Mol. Sci. 22 (4), 1564. 10.3390/ijms22041564 33557244 PMC7913895

[B96] Inesi G. de Meis L. (1989). Regulation of steady state filling in sarcoplasmic reticulum: Roles of back-inhibition, leakage, and slippage of the calcium pump. J. Biol. Chem. 264 (10), 5929–5936. 10.1016/s0021-9258(18)83639-0 2522442

[B97] Ioannou M. Coutsogeorgopoulos C. Synetos D. (1998). Kinetics of inhibition of rabbit reticulocyte peptidyltransferase by anisomycin and sparsomycin. Mol. Pharmacol. 53 (6), 1089–1096. 9614213

[B98] Iordanov M. S. Pribnow D. Magun J. L. Dinh T. H. Pearson J. A. Chen S. L. (1997). Ribotoxic stress response: Activation of the stress-activated protein kinase JNK1 by inhibitors of the peptidyl transferase reaction and by sequence-specific RNA damage to the α-sarcin/ricin loop in the 28S rRNA. Mol. Cell. Biol. 17 (6), 3373–3381. 10.1128/mcb.17.6.3373 9154836 PMC232190

[B99] Ishihara H. Asano T. Tsukuda K. Katagiri H. Inukai K. Anai M. (1993). Pancreatic β cell line MIN6 exhibits characteristics of glucose metabolism and glucose-stimulated insulin secretion similar to those of normal islets. Diabetologia 36 (11), 1139–1145. 10.1007/BF00401058 8270128

[B100] Jung S. J. Kim H. (2021). Emerging view on the molecular functions of Sec62 and Sec63 in protein translocation. Int. J. Mol. Sci. 22 (23), 12757. 10.3390/ijms222312757 34884562 PMC8657602

[B101] Kelleher D. J. Gilmore R. (2006). An evolving view of the eukaryotic oligosaccharyltransferase. Glycobiology 16 (4), 47R–62R. 10.1093/glycob/cwj066 16317064

[B102] Khan M. A. B. Hashim M. J. King J. K. Govender R. D. Mustafa H. Al Kaabi J. (2020). Epidemiology of type 2 diabetes – global burden of disease and forecasted trends. J. Epidemiol. Glob. Health 10 (1), 107–111. 10.2991/jegh.k.191028.001 32175717 PMC7310804

[B103] Kim S. Kim D. K. Jeong S. Lee J. (2022). The common cellular events in the neurodegenerative diseases and the associated role of endoplasmic reticulum stress. Int. J. Mol. Sci. 23 (11), 5894. 10.3390/ijms23115894 35682574 PMC9180188

[B104] Kiviluoto S. Vervliet T. Ivanova H. Decuypere J. P. De Smedt H. Missiaen L. (2013). Regulation of inositol 1, 4, 5-trisphosphate receptors during endoplasmic reticulum stress. Biochim. Biophys. Acta 1833 (7), 1612–1624. 10.1016/j.bbamcr.2013.01.026 23380704

[B105] Klec C. Madreiter-Sokolowski C. T. Stryeck S. Sachdev V. Duta-Mare M. Gottschalk B. (2019). Glycogen synthase kinase 3β controls presenilin-1-mediated endoplasmic reticulum Ca^2+^ leak directed to mitochondria in pancreatic islets and β-cells. Cell. Physiol. biochem. 52 (1), 57–75. 10.33594/000000005 30790505 PMC6459368

[B106] Klein M. C. Zimmermann K. Schorr S. Landini M. Klemens P. A. W. Altensell J. (2018). AXER is an ATP/ADP exchanger in the membrane of the endoplasmic reticulum. Nat. Commun. 9 (1), 3489. 10.1038/s41467-018-06003-9 30154480 PMC6113206

[B107] Konieczny V. Keebler M. V. Taylor C. W. (2012). Spatial organization of intracellular Ca^2+^ signals. Semin. Cell Dev. Biol. 23 (2), 172–180. 10.1016/j.semcdb.2011.09.006 21925615

[B108] Kovacs G. Reimer L. Jensen P. H. (2021). Endoplasmic reticulum-based calcium dysfunctions in synucleinopathies. Front. Neurol. 12, 742625. 10.3389/fneur.2021.742625 34744980 PMC8563702

[B109] Krebs J. Agellon L. B. Michalak M. (2015). Ca^2+^ homeostasis and endoplasmic reticulum (ER) stress: An integrated view of calcium signaling. Biochem. Biophys. Res. Commun. 460 (1), 114–121. 10.1016/j.bbrc.2015.02.004 25998740

[B110] Kriegler T. Kiburg G. Hessa T. (2020). Translocon-associated protein complex (TRAP) is crucial for co-translational translocation of pre-proinsulin. J. Mol. Biol. 432 (24), 166694. 10.1016/j.jmb.2020.10.028 33137310

[B111] La Rovere R. M. L. Roest G. Bultynck G. Parys J. B. (2016). Intracellular Ca^2+^ signaling and Ca^2+^ microdomains in the control of cell survival, apoptosis and autophagy. Cell Calcium 60 (2), 74–87. 10.1016/j.ceca.2016.04.005 27157108

[B112] Ladiges W. C. Knoblaugh S. E. Morton J. F. Korth M. J. Sopher B. L. Baskin C. R. (2005). Pancreatic β-cell failure and diabetes in mice with a deletion mutation of the endoplasmic reticulum molecular chaperone gene P58IPK. Diabetes 54 (4), 1074–1081. 10.2337/diabetes.54.4.1074 15793246

[B113] Lakkaraju A. K. Abrami L. Lemmin T. Blaskovic S. Kunz B. Kihara A. (2012). Palmitoylated calnexin is a key component of the ribosome–translocon complex. EMBO J. 31 (7), 1823–1835. 10.1038/emboj.2012.15 22314232 PMC3321195

[B114] Lam A. K. M. Galione A. (2013). The endoplasmic reticulum and junctional membrane communication during calcium signaling. Biochim. Biophys. Acta 1833 (11), 2542–2559. 10.1016/j.bbamcr.2013.06.004 23770047

[B115] Lang S. Benedix J. Fedeles S. V. Schorr S. Schirra C. Schäuble N. (2012). Different effects of Sec61α, Sec62 and Sec63 depletion on transport of polypeptides into the endoplasmic reticulum of mammalian cells. J. Cell Sci. 125 (8), 1958–1969. 10.1242/jcs.096727 22375059 PMC4074215

[B116] Lang S. Nguyen D. Pfeffer S. Förster F. Helms V. Zimmermann R. (2019). Functions and mechanisms of the human ribosome-translocon complex. Subcell. Biochem. 93, 83–141. 10.1007/978-3-030-28151-9_4 31939150

[B117] Lang S. Pfeffer S. Lee P. H. Cavalié A. Helms V. Förster F. (2017). An update on Sec61 channel functions, mechanisms, and related diseases. Front. Physiol. 8, 887. 10.3389/fphys.2017.00887 29163222 PMC5672155

[B118] Lanner J. T. (2012). Ryanodine receptor physiology and its role in disease. Adv. Exp. Med. Biol. 740, 217–234. 10.1007/978-94-007-2888-2_9 22453944

[B119] Layhadi J. A. Fountain S. J. (2017). Influence of ER leak on resting cytoplasmic Ca^2+^ and receptor-mediated Ca^2+^ signalling in human macrophage. Biochem. Biophys. Res. Commun. 487 (3), 633–639. 10.1016/j.bbrc.2017.04.106 28435065

[B120] Leamy A. K. Egnatchik R. A. Shiota M. Ivanova P. T. Myers D. S. Brown H. A. (2014). Enhanced synthesis of saturated phospholipids is associated with ER stress and lipotoxicity in palmitate treated hepatic cells. J. Lipid Res. 55 (7), 1478–1488. 10.1194/jlr.M050237 24859739 PMC4076085

[B121] Lebeaupin C. Vallée D. Hazari Y. Hetz C. Chevet E. Bailly-Maitre B. (2018). Endoplasmic reticulum stress signalling and the pathogenesis of non-alcoholic fatty liver disease. J. Hepatol. 69 (4), 927–947. 10.1016/j.jhep.2018.06.008 29940269

[B122] Lemos F. O. Bultynck G. Parys J. B. (2021). A comprehensive overview of the complex world of the endo- and sarcoplasmic reticulum Ca^2+^-leak channels. Biochim. Biophys. Acta. Mol. Cell Res. 1868 (7), 119020. 10.1016/j.bbamcr.2021.119020 33798602

[B123] Li L. Park E. Ling J. Ingram J. Ploegh H. Rapoport T. A. (2016). Crystal structure of a substrate-engaged SecY protein-translocation channel. Nature 531 (7594), 395–399. 10.1038/nature17163 26950603 PMC4855518

[B124] Lièvremont J. P. Rizzuto R. Hendershot L. Meldolesi J. (1997). BiP, a major chaperone protein of the endoplasmic reticulum lumen, plays a direct and important role in the storage of the rapidly exchanging pool of Ca^2+^ . J. Biol. Chem. 272 (49), 30873–30879. 10.1074/jbc.272.49.30873 9388233

[B125] Lim D. Dematteis G. Tapella L. Genazzani A. A. Calì T. Brini M. (2021). Ca^2+^ handling at the mitochondria-ER contact sites in neurodegeneration. Cell Calcium 98, 102453. 10.1016/j.ceca.2021.102453 34399235

[B126] Linxweiler M. Schorr S. Schäuble N. Jung M. Linxweiler J. Langer F. (2013). Targeting cell migration and the endoplasmic reticulum stress response with calmodulin antagonists: A clinically tested small molecule phenocopy of SEC62 gene silencing in human tumor cells. BMC Cancer 13, 574. 10.1186/1471-2407-13-574 24304694 PMC3878975

[B127] Liu E. A. Lieberman A. P. (2019). The intersection of lysosomal and endoplasmic reticulum calcium with autophagy defects in lysosomal diseases. Neurosci. Lett. 697, 10–16. 10.1016/j.neulet.2018.04.049 29704574 PMC6202281

[B128] Lloyd D. J. Wheeler M. C. Gekakis N. (2010). A point mutation in Sec61alpha1 leads to diabetes and hepatosteatosis in mice. Diabetes 59 (2), 460–470. 10.2337/db08-1362 19934005 PMC2809972

[B129] Lloyd-Evans E. Platt F. M. (2011). Lysosomal Ca^2+^ homeostasis: Role in pathogenesis of lysosomal storage diseases. Cell Calcium 50 (2), 200–205. 10.1016/j.ceca.2011.03.010 21724254

[B130] Lomax R. B. Camello C. Van Coppenolle F. Petersen O. H. Tepikin A. V. (2002). Basal and physiological Ca^2+^ leak from the endoplasmic reticulum of pancreatic acinar cells. Second messenger-activated channels and translocons. J. Biol. Chem. 277 (29), 26479–26485. 10.1074/jbc.M201845200 11994289

[B131] Luo T. Kim J. K. Chen B. Abdel-Latif A. Kitakaze M. Yan L. (2015). Attenuation of ER stress prevents post-infarction-induced cardiac rupture and remodeling by modulating both cardiac apoptosis and fibrosis. Chem. Biol. Interact. 225, 90–98. 10.1016/j.cbi.2014.10.032 25450231 PMC4684183

[B132] Ma Y. Hendershot L. M. (2001). The unfolding tale of the unfolded protein response. Cell 107 (7), 827–830. 10.1016/s0092-8674(01)00623-7 11779459

[B133] Mahamid J. Pfeffer S. Schaffer M. Villa E. Danev R. Kuhn Cuellar L. (2016). Visualizing the molecular sociology at the HeLa cell nuclear periphery. Science 351 (6276), 969–972. 10.1126/science.aad8857 26917770

[B134] Mandic A. Hansson J. Linder S. Shoshan M. C. (2003). Cisplatin induces endoplasmic reticulum stress and nucleus-independent apoptotic signaling. J. Biol. Chem. 278 (11), 9100–9106. 10.1074/jbc.M210284200 12509415

[B135] Mariángelo J. I. E. Román B. Silvestri M. A. Salas M. Vittone L. Said M. (2020). Chemical chaperones improve the functional recovery of stunned myocardium by attenuating the endoplasmic reticulum stress. Acta Physiol. 228 (2), e13358. 10.1111/apha.13358 31385408

[B136] Mariángelo J. I. E. Valverde C. A. Vittone L. Said M. Mundiña-Weilenmann C. (2022). Pharmacological inhibition of translocon is sufficient to alleviate endoplasmic reticulum stress and improve Ca^2+^ handling and contractile recovery of stunned myocardium. Eur. J. Pharmacol. 914, 174665. 10.1016/j.ejphar.2021.174665 34861208

[B137] Martino L. Masini M. Novelli M. Beffy P. Bugliani M. Marselli L. (2012). Palmitate activates autophagy in INS-1E β-cells and in isolated rat and human Pancreatic Islets. PLoS One 7 (5), e36188. 10.1371/journal.pone.0036188 22563482 PMC3341371

[B138] Minamino T. Kitakaze M. (2010). ER stress in cardiovascular disease. J. Mol. Cell. Cardiol. 48 (6), 1105–1110. 10.1016/j.yjmcc.2009.10.026 19913545

[B139] Miyazaki J. I. Araki K. Yamato E. Ikegami H. Asano T. Shibasaki Y. (1990). Establishment of a pancreatic β cell line that retains glucose-inducible insulin secretion: Special reference to expression of glucose transporter isoforms. Endocrinology 127 (1), 126–132. 10.1210/endo-127-1-126 2163307

[B140] Mohanty S. P Chaudhary B. Zoetewey D. (2020). Structural Insight into the mechanism of N-linked glycosylation by oligosaccharyltransferase. Biomolecules 10 (4), 624. 10.3390/biom10040624 PMC7226087 32316603

[B141] Mohorko E. Owen R. L. Malojčić G. Brozzo M. S. Aebi M. Glockshuber R. (2014). Structural basis of substrate specificity of human oligosaccharyl transferase subunit N33/Tusc3 and its role in regulating protein N-glycosylation. Structure 22 (4), 590–601. 10.1016/j.str.2014.02.013 24685145

[B142] Morel J. D. Paatero A. O. Wei J. Yewdell J. W. Guenin-Macé L. Van Haver D. (2018). Proteomics reveals scope of mycolactone-mediated Sec61 blockade and distinctive stress signature. Mol. Cell. Proteomics 17 (9), 1750–1765. 10.1074/mcp.RA118.000824 29915147 PMC6126388

[B143] Müller L. de Escauriaza M. D. Lajoie P. Theis M. Jung M. Müller A. (2010). Evolutionary gain of function for the ER membrane protein Sec62 from yeast to humans. Mol. Biol. Cell 21 (5), 691–703. 10.1091/mbc.e09-08-0730 20071467 PMC2828957

[B144] Needham P. G. Guerriero C. J. Brodsky J. L. (2019). Chaperoning endoplasmic reticulum–associated degradation (ERAD) and protein conformational diseases. Cold Spring Harb. Perspect. Biol. 11 (8), a033928. 10.1101/cshperspect.a033928 30670468 PMC6671943

[B145] Nguyen D. Stutz R. Schorr S. Lang S. Pfeffer S. Freeze H. H. (2018). Proteomics reveals signal peptide features determining the client specificity in human TRAP-dependent ER protein import. Nat. Commun. 9, 3765. 10.1038/s41467-018-06188-z 30217974 PMC6138672

[B146] Ni M. Zhang Y. Lee A. S. (2011). Beyond the endoplasmic reticulum: Atypical GRP78 in cell viability, signalling and therapeutic targeting. Biochem. J. 434 (2), 181–188. 10.1042/BJ20101569 21309747 PMC3353658

[B147] Nicchitta C. V. Zheng T. (1997). Regulation of the ribosome–membrane junction at early stages of presecretory protein translocation in the mammalian endoplasmic reticulum. J. Cell Biol. 139 (7), 1697–1708. 10.1083/jcb.139.7.1697 9412465 PMC2132637

[B148] O’Keefe S. High S. Demangel C. (2022). Biochemical and biological assays of mycolactone-mediated inhibition of Sec61. Methods Mol. Biol. 2387, 163–181. 10.1007/978-1-0716-1779-3_16 34643911

[B149] Oliver J. D. Hresko R. C. Mueckler M. High S. (1996). The Glut 1 glucose transporter interacts with calnexin and calreticulin. J. Biol. Chem. 271 (23), 13691–13696. 10.1074/jbc.271.23.13691 8662691

[B150] Oliver J. D. van der Wal F. J. Bulleid N. J. High S. (1997). Interaction of the thiol-dependent reductase ERp57 with nascent glycoproteins. Science 275 (5296), 86–88. 10.1126/science.275.5296.86 8974399

[B151] Ong H. L. Liu X. Sharma A. Hegde R. S. Ambudkar I. S. (2007). Intracellular Ca^2+^ release via the ER translocon activates store-operated calcium entry. Pflugers Arch. 453 (6), 797–808. 10.1007/s00424-006-0163-5 17171366

[B152] Orrenius S. Zhivotovsky B. Nicotera P. (2003). Regulation of cell death: The calcium–apoptosis link. Nat. Rev. Mol. Cell Biol. 4 (7), 552–565. 10.1038/nrm1150 12838338

[B153] Ovize M. Baxter G. F. Di Lisa F. Ferdinandy P. Garcia-Dorado D. Hausenloy D. J. (2010). Postconditioning and protection from reperfusion injury: Where do we stand? Position Paper from the working group of cellular biology of the heart of the European Society of Cardiology. Cardiovasc. Res. 87 (3), 406–423. 10.1093/cvr/cvq129 20448097

[B154] Özcan U. Yilmaz E. Özcan L. Furuhashi M. Vaillancourt E. Smith R. O. (2006). Chemical chaperones reduce ER stress and restore glucose homeostasis in a mouse model of type 2 diabetes. Science 313 (5790), 1137–1140. 10.1126/science.1128294 16931765 PMC4741373

[B155] Papa F. R. (2012). Endoplasmic reticulum stress, pancreatic β-cell degeneration, and diabetes. Cold Spring Harb. Perspect. Med. 2 (9), a007666. 10.1101/cshperspect.a007666 22951443 PMC3426819

[B156] Park E. Ménétret J. F. Gumbart J. C. Ludtke S. J. Li W. Whynot A. (2014). Structure of the SecY channel during initiation of protein translocation. Nature 506 (7486), 102–106. 10.1038/nature12720 24153188 PMC3948209

[B157] Pestova T. V. Hellen C. U. (2001). Functions of eukaryotic factors in initiation of translation. Cold Spring Harb. Symp. Quant. Biol. 66, 389–396. 10.1101/sqb.2001.66.389 12762041

[B158] Peters L. R. Raghavan M. (2011). Endoplasmic reticulum calcium depletion impacts chaperone secretion, innate immunity, and phagocytic uptake of cells. J. Immunol. 187 (2), 919–931. 10.4049/jimmunol.1100690 21670312 PMC3371385

[B159] Pfeffer S. Burbaum L. Unverdorben P. Pech M. Chen Y. Zimmermann R. (2015). Structure of the native Sec61 protein-conducting channel. Nat. Commun. 6, 8403. 10.1038/ncomms9403 26411746 PMC4598622

[B160] Pfeffer S. Dudek J. Gogala M. Schorr S. Linxweiler J. Lang S. (2014). Structure of the mammalian oligosaccharyl-transferase complex in the native ER protein translocon. Nat. Commun. 5 (1), 3072. 10.1038/ncomms4072 24407213

[B161] Pinton P. Giorgi C. Siviero R. Zecchini E. Rizzuto R. (2008). Calcium and apoptosis: ER-mitochondria Ca^2+^ transfer in the control of apoptosis. Oncogene 27 (50), 6407–6418. 10.1038/onc.2008.308 18955969 PMC2844952

[B162] Pobre K. F. R. Poet G. J. Hendershot L. M. (2019). The endoplasmic reticulum (ER) chaperone BiP is a master regulator of ER functions: Getting by with a little help from ERdj friends. J. Biol. Chem. 294 (6), 2098–2108. 10.1074/jbc.REV118.002804 30563838 PMC6369273

[B163] Prole D. L. Taylor C. W. (2016). Inositol 1, 4, 5‐trisphosphate receptors and their protein partners as signalling hubs. J. Physiol. 594 (11), 2849–2866. 10.1113/JP271139 26830355 PMC4887697

[B164] Prole D. L. Taylor C. W. (2019). Structure and function of IP3 receptors. Cold Spring Harb. Perspect. Biol. 11 (4), a035063. 10.1101/cshperspect.a035063 30745293 PMC6442203

[B165] Raden D. Song W. Gilmore R. (2000). Role of the cytoplasmic segments of Sec61alpha in the ribosome-binding and translocation-promoting activities of the Sec61 complex. J. Cell Biol. 150 (1), 53–64. 10.1083/jcb.150.1.53 10893256 PMC2185549

[B166] Ramachandra C. J. A. Hernandez-Resendiz S. Crespo-Avilan G. E. Lin Y. H. Hausenloy D. J. (2020). Mitochondria in acute myocardial infarction and cardioprotection. EBioMedicine 57, 102884. 10.1016/j.ebiom.2020.102884 32653860 PMC7355051

[B167] Ramírez A. S. Kowal J. Locher K. P. (2019). Cryo–electron microscopy structures of human oligosaccharyltransferase complexes OST-A and OST-B. Science 366 (6471), 1372–1375. 10.1126/science.aaz3505 31831667

[B168] Reddy R. K. Mao C. Baumeister P. Austin R. C. Kaufman R. J. Lee A. S. (2003). Endoplasmic reticulum chaperone protein GRP78 protects cells from apoptosis induced by topoisomerase inhibitors: Role of ATP binding site in suppression of caspase-7 activation. J. Biol. Chem. 278 (23), 20915–20924. 10.1074/jbc.M212328200 12665508

[B169] Ren H. Zhai W. Lu X. Wang G. (2021). The cross-Links of endoplasmic reticulum stress, autophagy, and neurodegeneration in Parkinson's disease. Front. Aging Neurosci. 13, 691881. 10.3389/fnagi.2021.691881 34168552 PMC8218021

[B170] Risérus U. Willett W. C. Hu F. B. (2009). Dietary fats and prevention of type 2 diabetes. Prog. Lipid Res. 48 (1), 44–51. 10.1016/j.plipres.2008.10.002 19032965 PMC2654180

[B171] Rodriguez-Fonseca C. Amils R. Garrett R. A. (1995). Fine structure of the peptidyl transferase centre on 23 S-like rRNAs deduced from chemical probing of antibiotic-ribosome complexes. J. Mol. Biol. 247 (2), 224–235. 10.1006/jmbi.1994.0135 7707371

[B172] Römisch K. (2017). A case for Sec61 channel involvement in ERAD. Trends biochem. Sci. 42 (3), 171–179. 10.1016/j.tibs.2016.10.005 27932072

[B173] Ron D. Walter P. (2007). Signal integration in the endoplasmic reticulum unfolded protein response. Nat. Rev. Mol. Cell Biol. 8 (7), 519–529. 10.1038/nrm2199 17565364

[B174] Roy A. Wonderlin W. F. (2003). The permeability of the endoplasmic reticulum is dynamically coupled to protein synthesis. J. Biol. Chem. 278 (7), 4397–4403. 10.1074/jbc.M207295200 12458217

[B175] Ruiz-Canada C. Kelleher D. J. Gilmore R. (2009). Cotranslational and posttranslational N-glycosylation of polypeptides by distinct mammalian OST isoforms. Cell 136 (2), 272–283. 10.1016/j.cell.2008.11.047 19167329 PMC2859625

[B176] Sammels E. Parys J. B. Missiaen L. De Smedt H. Bultynck G. (2010). Intracellular Ca^2+^ storage in health and disease: A dynamic equilibrium. Cell Calcium 47 (4), 297–314. 10.1016/j.ceca.2010.02.001 20189643

[B177] Sarfo F. S. Phillips R. Wansbrough-Jones M. Simmonds R. E. (2016). Recent advances: Role of mycolactone in the pathogenesis and monitoring of Mycobacterium ulcerans infection/Buruli ulcer disease. Cell. Microbiol. 18 (1), 17–29. 10.1111/cmi.12547 26572803 PMC4705457

[B178] Schäuble N. Lang S. Jung M. Cappel S. Schorr S. Ulucan Ö. (2012). BiP-mediated closing of the Sec61 channel limits Ca^2+^ leakage from the ER. EMBO J. 31 (15), 3282–3296. 10.1038/emboj.2012.189 22796945 PMC3411083

[B179] Schnell D. J. Hebert D. N. (2003). Protein translocons: Multifunctional mediators of protein translocation across membranes. Cell 112 (4), 491–505. 10.1016/s0092-8674(03)00110-7 12600313

[B180] Schoebel S. Mi W. Stein A. Ovchinnikov S. Pavlovicz R. DiMaio F. (2017). Cryo-EM structure of the protein-conducting ERAD channel Hrd1 in complex with Hrd3. Nature 548 (7667), 352–355. 10.1038/nature23314 28682307 PMC5736104

[B181] Schorr S. Klein M. C. Gamayun I. Melnyk A. Jung M. Schäuble N. (2015). Co-chaperone specificity in gating of the polypeptide conducting channel in the membrane of the human endoplasmic reticulum. J. Biol. Chem. 290 (30), 18621–18635. 10.1074/jbc.M115.636639 26085089 PMC4513120

[B182] Schubert D. Klein M. C. Hassdenteufel S. Caballero-Oteyza A. Yang L. Proietti M. (2018). Plasma cell deficiency in human subjects with heterozygous mutations in Sec61 translocon alpha 1 subunit (SEC61A1). J. Allergy Clin. Immunol. 141 (4), 1427–1438. 10.1016/j.jaci.2017.06.042 28782633 PMC5797495

[B183] Shacham T. Sharma N. Lederkremer G. Z. (2019). Protein misfolding and ER stress in Huntington’s disease. Front. Mol. Biosci. 6, 20. 10.3389/fmolb.2019.00020 31001537 PMC6456712

[B184] Shen K. Arslan S. Akopian D. Ha T. Shan S. O. (2012). Activated GTPase movement on an RNA scaffold drives cotranslational protein targeting. Nature 492 (7428), 271–275. 10.1038/nature11726 23235881 PMC3531814

[B185] Shilling D. Mak D. O. D. Kang D. E. Foskett J. K. (2012). Lack of evidence for presenilins as endoplasmic reticulum Ca^2+^ leak channels. J. Biol. Chem. 287 (14), 10933–10944. 10.1074/jbc.M111.300491 22311977 PMC3322867

[B186] Shilling D. Müller M. Takano H. Mak D. O. D. Abel T. Coulter D. A. (2014). Suppression of InsP3 receptor-mediated Ca^2+^ signaling alleviates mutant presenilin-linked familial Alzheimer’s disease pathogenesis. J. Neurosci. 34 (20), 6910–6923. 10.1523/JNEUROSCI.5441-13.2014 24828645 PMC4019804

[B187] Sicking M. Lang S. Bochen F. Roos A. Drenth J. P. H. Zakaria M. (2021). Complexity and specificity of Sec61-channelopathies: Human diseases affecting gating of the Sec61 complex. Cells 10 (5), 1036. 10.3390/cells10051036 33925740 PMC8147068

[B188] Silberstein S. Gilmore R. (1996). Biochemistry, molecular biology, and genetics of the oligosaccharyltransferase. FASEB J. 10 (8), 849–858. 10.1096/fasebj.10.8.8666161 8666161

[B189] Simon S. M. Blobel G. (1991). A protein-conducting channel in the endoplasmic reticulum. Cell 65 (3), 371–380. 10.1016/0092-8674(91)90455-8 1902142

[B190] Sommer N. Junne T. Kalies K. U. Spiess M. Hartmann E. (2013). TRAP assists membrane protein topogenesis at the mammalian ER membrane. Biochim. Biophys. Acta 1833 (12), 3104–3111. 10.1016/j.bbamcr.2013.08.018 24013069

[B191] Song B. Scheuner D. Ron D. Pennathur S. Kaufman R. J. (2008). CHOP deletion reduces oxidative stress, improves β cell function, and promotes cell survival in multiple mouse models of diabetes. J. Clin. Invest. 118 (10), 3378–3389. 10.1172/JCI34587 18776938 PMC2528909

[B192] Song W. Raden D. Mandon E. C. Gilmore R. (2000). Role of Sec61alpha in the regulated transfer of the ribosome-nascent chain complex from the signal recognition particle to the translocation channel. Cell 100 (3), 333–343. 10.1016/s0092-8674(00)80669-8 10676815

[B193] Swanton E. Bulleid N. J. (2003). Protein folding and translocation across the endoplasmic reticulum membrane. Mol. Membr. Biol. 20 (2), 99–104. 10.1080/0968768031000069241 12851067

[B194] Synofzik M. Haack T. B. Kopajtich R. Gorza M. Rapaport D. Greiner M. (2014). Absence of BiP co-chaperone DNAJC3 causes diabetes mellitus and multisystemic neurodegeneration. Am. J. Hum. Genet. 95 (6), 689–697. 10.1016/j.ajhg.2014.10.013 25466870 PMC4259973

[B195] Takeshima H. Venturi E. Sitsapesan R. (2015). New and notable ion-channels in the sarcoplasmic/endoplasmic reticulum: Do they support the process of intracellular Ca^2+^ release? J. Physiol. 593 (15), 3241–3251. 10.1113/jphysiol.2014.281881 26228553 PMC4553049

[B196] Tatu U. Helenius A. (1997). Interactions between newly synthesized glycoproteins, calnexin and a network of resident chaperones in the endoplasmic reticulum. J. Cell Biol. 136 (3), 555–565. 10.1083/jcb.136.3.555 9024687 PMC2134297

[B197] Traut R. R. Monro R. E. (1964). The puromycin reaction and its relation to protein synthesis. J. Mol. Biol. 10 (1), 63–72. 10.1016/s0022-2836(64)80028-0 14222897

[B198] Tsai Y. C. Weissman A. M. (2010). The unfolded protein response, degradation from endoplasmic reticulum and cancer. Genes Cancer 1 (7), 764–778. 10.1177/1947601910383011 21331300 PMC3039444

[B199] Tu H. Nelson O. Bezprozvanny A. Wang Z. Lee S. F. Hao Y. H. (2006). Presenilins form ER Ca^2+^ leak channels, a function disrupted by familial Alzheimer’s disease-linked mutations. Cell 126 (5), 981–993. 10.1016/j.cell.2006.06.059 16959576 PMC3241869

[B200] Tyedmers J. Lerner M. Bies C. Dudek J. Skowronek M. H. Haas I. G. (2000). Homologs of the yeast Sec complex subunits Sec62p and Sec63p are abundant proteins in dog pancreas microsomes. Proc. Natl. Acad. Sci. U. S. A. 97 (13), 7214–7219. 10.1073/pnas.97.13.7214 10860986 PMC16525

[B201] Ureshino R. P. Erustes A. G. Bassani T. B. Wachilewski P. Guarache G. C. Nascimento A. C. (2019). The interplay between Ca^2+^ signaling pathways and neurodegeneration. Int. J. Mol. Sci. 20 (23), 6004. 10.3390/ijms20236004 PMC6928941 31795242

[B202] Van Coppenolle F. Vanden Abeele F. Slomianny C. Flourakis M. Hesketh J. Dewailly E. (2004). Ribosome-translocon complex mediates calcium leakage from endoplasmic reticulum stores. J. Cell Sci. 117 (18), 4135–4142. 10.1242/jcs.01274 15280427

[B203] Van Nieuwenhove E. Barber J. S. Neumann J. Smeets E. Willemsen M. Pasciuto E. (2020). Defective Sec61α1 underlies a novel cause of autosomal dominant severe congenital neutropenia. J. Allergy Clin. Immunol. 146 (5), 1180–1193. 10.1016/j.jaci.2020.03.034 32325141 PMC7649975

[B204] Vanderheyden V. Devogelaere B. Missiaen L. De Smedt H. Bultynck G. Parys J. B. (2009). Regulation of inositol 1, 4, 5-trisphosphate-induced Ca^2+^ release by reversible phosphorylation and dephosphorylation. Biochim. Biophys. Acta 1793 (6), 959–970. 10.1016/j.bbamcr.2008.12.003 19133301 PMC2693466

[B205] Virrey J. J. Dong D. Stiles C. Patterson J. B. Pen L. Ni M. (2008). Stress chaperone GRP78/BiP confers chemoresistance to tumor-associated endothelial cells. Mol. Cancer Res. 6 (8), 1268–1275. 10.1158/1541-7786.MCR-08-0060 18708359 PMC2593417

[B206] von Heijne G. Gavel Y. (1988). Topogenic signals in integral membrane proteins. Eur. J. Biochem. 174 (4), 671–678. 10.1111/j.1432-1033.1988.tb14150.x 3134198

[B207] Voorhees R. M. Fernández I. S. Scheres S. H. W. Hegde R. S. (2014). Structure of the mammalian ribosome-Sec61 complex to 3.4 Å resolution. Cell 157 (7), 1632–1643. 10.1016/j.cell.2014.05.024 24930395 PMC4081569

[B208] Voorhees R. M. Hegde R. S. (2016). Structure of the Sec61 channel opened by a signal sequence. Science 351 (6268), 88–91. 10.1126/science.aad4992 26721998 PMC4700591

[B209] Wacquier B. Voorsluijs V. Combettes L. Dupont G. (2019). Coding and decoding of oscillatory Ca^2+^ signals. Semin. Cell Dev. Biol. 94, 11–19. 10.1016/j.semcdb.2019.01.008 30659886

[B210] Wang L. Dobberstein B. (1999). Oligomeric complexes involved in translocation of proteins across the membrane of the endoplasmic reticulum. FEBS Lett. 457 (3), 316–322. 10.1016/s0014-5793(99)01075-3 10471800

[B211] Wang Q. Li L. Ye Y. (2008). Inhibition of p97-dependent protein degradation by eeyarestatin I. J. Biol. Chem. 283 (12), 7445–7454. 10.1074/jbc.M708347200 18199748 PMC2276333

[B212] Wang Q. Mora-Jensen H. Weniger M. A. Perez-Galan P. Wolford C. Hai T. (2009). ERAD inhibitors integrate ER stress with an epigenetic mechanism to activate BH3-only protein NOXA in cancer cells. Proc. Natl. Acad. Sci. U. S. A. 106 (7), 2200–2205. 10.1073/pnas.0807611106 19164757 PMC2629785

[B213] Wirth A. Jung M. Bies C. Frien M. Tyedmers J. Zimmermann R. (2003). The Sec61p complex is a dynamic precursor activated channel. Mol. Cell 12 (1), 261–268. 10.1016/s1097-2765(03)00283-1 12887911

[B214] Wonderlin W. F. (2009). Constitutive, translation-independent opening of the protein-conducting channel in the endoplasmic reticulum. Pflugers Arch. 457 (4), 917–930. 10.1007/s00424-008-0545-y 18604553

[B215] Wu X. Rapoport T. A. (2018). Mechanistic insights into ER-associated protein degradation. Curr. Opin. Cell Biol. 53, 22–28. 10.1016/j.ceb.2018.04.004 29719269 PMC6131047

[B216] Yasmeen N. Datta M. Kumar V. Alshehri F. S. Almalki A. H. Haque S. (2022). Deciphering the link between ER^UPR^ signaling and microRNA in pathogenesis of Alzheimer’s disease. Front. Aging Neurosci. 14, 880167. 10.3389/fnagi.2022.880167 35615589 PMC9126300

[B217] Yotsu R. R. Suzuki K. Simmonds R. E. Bedimo R. Ablordey A. Yeboah-Manu D. (2018). Buruli ulcer: A review of the current knowledge. Curr. Trop. Med. Rep. 5 (4), 247–256. 10.1007/s40475-018-0166-2 30460172 PMC6223704

[B218] Yusupov M. M. Yusupova G. Z. Baucom A. Lieberman K. Earnest T. N. Cate J. H. (2001). Crystal structure of the ribosome at 5.5 Å resolution. Science 292 (5518), 883–896. 10.1126/science.1060089 11283358

[B219] Zatti G. Burgo A. Giacomello M. Barbiero L. Ghidoni R. Sinigaglia G. (2006). Presenilin mutations linked to familial Alzheimer’s disease reduce endoplasmic reticulum and Golgi apparatus calcium levels. Cell Calcium 39 (6), 539–550. 10.1016/j.ceca.2006.03.002 16620965

[B220] Zhang I. X. Raghavan M. Satin L. S. (2019). The endoplasmic reticulum and calcium homeostasis in pancreatic β cells. Endocrinology 161 (2), bqz028. 10.1210/endocr/bqz028 PMC7028010 31796960

[B221] Zhang K. Kaufman R. J. (2008). From endoplasmic-reticulum stress to the inflammatory response. Nature 454 (7203), 455–462. 10.1038/nature07203 18650916 PMC2727659

